# Age-related changes in individuals with and without reading disability: Behavioral and fMRI evidence

**DOI:** 10.1162/imag_a_00232

**Published:** 2024-07-15

**Authors:** Xiaohui Yan, Guoyan Feng, Yang Fu, Jia Hua, Fan Cao

**Affiliations:** Department of Psychology, The University of Hong Kong, Hong Kong, China; State Key Lab of Brain and Cognitive Sciences, The University of Hong Kong; Department of Psychology, Sun Yat-Sen University, Guangzhou, China; School of Management, Guangzhou Xinhua University, Guangzhou, China; Instrumental Analysis and Research Center, Sun Yat-Sen University, Guangzhou, China

**Keywords:** reading disability, fMRI, development

## Abstract

Reading disability (RD) is a developmental neurological disorder with high prevalence across languages; however, the developmental differences in the behavior and brain of individuals with RD remain poorly understood, especially in Chinese RD. In the current study, we aimed to differentiate persistent deficits in Chinese children and adults with RD, differences that are evident only in children but not adults with RD, and differences that are more severe in adults than children with RD. In a cross-sectional design, we compared behavioral performances in a battery of reading tests and brain activities in three tasks in Chinese children (N = 83, mean age = 11) and adults (N = 94, mean age = 20) with and without RD. We found that phonological deficits were persistent across children and adults with RD, while deficits in word decoding accuracy were only evident in children but not adults with RD. Moreover, deficits in sentence reading fluency were more severe in adults than children with RD. In the brain, we found persistent reduction of brain activation in the left inferior parietal lobule (IPL), suggesting neural signature of RD. We found greater reduction of brain activation in the left inferior frontal gyrus (IFG) in children with RD than in adults with RD, suggesting a developmental delay and/or performance effect. On the other hand, a reduction of brain activation in the left inferior temporal gyrus (ITG) was more salient in adults with RD than in children with RD, due to greater developmental increase in typical readers than in RD readers, ultimately indicating accumulative effects of RD. The results were replicated in multiple tasks and samples. It contributes to advancing our understanding of the etiology and prognosis of RD. The findings also have practical implications in precise diagnosis and interventions for RD at different ages.

## Abbreviations

TP = temporoparietal region; OT = occipitotemporal region; IFG = inferior frontal gyrus; ITG = inferior temporal gyrus; STG = superior temporal gyrus; fMRI = functional magnetic resonance imaging; ANOVA = analysis of variance; ANCOVA = analysis of covariance.

## 1 Introduction

Reading disability (RD) is defined as reading failure despite adequate cognitive abilities, reading instructions, and learning motivations (Shaywitz et al., 1995). Children with RD show low word reading accuracy, slow reading speed, and poor phonological awareness across languages (Brunswick et al., 1999;Shaywitz et al., 1999). The behaviors associated with RD change with age (Shaywitz et al., 1999;Van Der Auwera et al., 2021); however, very few studies have focused on developmental differences in RD beyond the end of formal schooling.

For normal reading development, the three-stage reading development model proposed byFrith (1986)suggested that logographic reading, phonological reading, and orthographic reading occur in a specific order during development. With reading development, the task of reading transitions from “learning to read” to “reading to learn.” Research suggests a shift from phonological reading to orthographic reading during development with both behavioral (Peng et al., 1985;Song et al., 1995) and neuroimaging evidence (Cao et al., 2010;Martin et al., 2015;Pugh et al., 2000). However, individuals with RD do not follow the three reading development stages at the same pace as typical readers, and the phonological reading stage may take much longer to achieve, with most people never reaching the orthographic reading stage (Frith, 1986;Morken et al., 2017).

Among the limited number of studies on reading development in individuals with RD, some found that adults with RD continue to show phonological awareness deficits (Bruck, 1992;Del Tufo & Earle, 2020;Vukovic et al., 2004) and slow reading speed (Carioti et al., 2021), despite their improved word reading accuracy over time. These findings suggest that phonological deficits are persistent in individuals with RD. Phonological deficits cause word decoding deficits in the early stages of reading acquisition because the lack of high-quality phoneme representations hinders the mapping between graphemes and phonemes. When reading becomes simply a means to learn, rapid word recognition based on the high quality of and strong connections between lexical representations is necessary for fluent reading. Therefore, although individuals with RD eventually develop mappings between graphemes and phonemes, they may still show significant impairment in fluent reading in adulthood due to deficient rapid word recognition and poor quality of lexical representations (Martin et al., 2015).

Even less attention has been given to the developmental differences in the brain in individuals with RD.Pugh et al. (2000)have argued that phonological deficits associated with the temporoparietal area cause later deficits in orthographic acquisition probably in the fusiform gyrus in individuals with RD. Therefore, there may be greater abnormalities in the dorsal phonological pathway in children with RD and greater abnormalities in the ventral orthographic pathway in adults with RD. Consistent with this hypothesis, in a meta-analysis of neuroimaging studies, brain activation differences in children and adults with RD were directly compared, and it was found that children showed greater reduction in the bilateral inferior parietal lobule (IPL) and that adults showed greater reduction of brain activation in the left fusiform gyrus (Richlan et al., 2011). Moreover, a whole-brain functional connectivity study found greater abnormalities in the connections with the occipitotemporal area in adults with RD than in children with RD (Finn et al., 2014), supporting the hypothesis.

Atypical reading development may be accompanied by an unsuccessful shift from dorsal phonological reading to ventral orthographic reading in the brain. One study examined age-related changes in brain activation in English speakers with and without RD for the age range of 7–18 years (Shaywitz et al., 2007). It was found that there was a positive correlation between age and brain activation in the left occipitotemporal (OT) area for typical readers and in the left inferior frontal gyrus (IFG) for RD readers, suggesting an increased involvement of the orthographic reading pathway with age in typical readers but not in RD readers. Another study longitudinally examined children with RD from 6 to 8 years old, and found that differences in the left vOT became more pronounced with age (Chyl et al., 2019), presumably due to greater development in this region in typical children than in RD children. Taken together, more research is needed to examine how reading develops in the atypical brain without the influence of intensive reading interventions, and that is the purpose of the current study.

Furthermore, previous developmental studies were almost all in alphabetic languages and no evidence is available from non-alphabetic languages in terms of how reading development takes place in people with RD behaviorally and neurologically. For example, not a single study has been published to examine brain differences in Chinese adults with RD. Chinese, as a morpho-syllabic language, does not have grapheme-phoneme-correspondence rules, and each character represents a syllable and a morpheme. Phonological decoding in Chinese relies on whole-character-to-whole-syllable mapping. Due to different phonological decoding procedures, the phonological reading pathway seems to be in different parts of the brain for people who speak various languages (Fiez, 2000;Paulesu et al., 2000). Previous studies have shown that while phonological assembly is associated with the left temporoparietal area in English speakers, Chinese word reading is associated with greater activation in the left dorsal IFG due to whole-character-to-whole-syllable mapping (Bolger et al., 2005;Tan et al., 2005). Furthermore, recent meta-analysis and reviews suggest greater structural and functional differences in the left dorsal IFG in Chinese readers with RD than in RD readers of alphabetic languages (Richlan, 2020;Yan et al., 2021). Therefore, we may observe developmental differences in different parts of the brain in Chinese compared to alphabetic languages.

One challenge in neuroimaging research is explaining the nature of the brain differences associated with RD. In the present study, from a developmental perspective, we hypothesized that there are three types of brain differences: (1) persistent brain differences in children and adults with RD across multiple tasks, representing the definitive signature associated with RD; (2) brain differences that exist only in children but not in adults, representing developmental delays/performance effects; and (3) brain differences that are more salient in adults than children with RD, representing the accumulative effects of RD. We expected that phonological awareness deficits are persistent in individuals with RD, while word decoding accuracy is a major deficit in children with RD and reading fluency is a major deficit in adults with RD. In the brain, we expected greater differences in the dorsal phonological reading pathway (i.e., the left dorsal IFG) in children than adults with RD, and greater differences in the ventral orthographic reading pathway (i.e., the left occipitotemporal area) in adults than children with RD. In other words, we expected greater developmental increase in RD readers in the left dorsal IFG for phonological reading and greater developmental increase in the left OT in typical readers for orthographic reading. We also expected persistent differences in the left temporoparietal area due to persistent phonological awareness deficits, suggesting neural signatures of RD.

We employed a 2 group (RD, age controls [ACs]) by 2 age (adults, children) experimental design, obtaining both behavioral and fMRI data. During the fMRI data acquisition, participants completed three different tasks, namely, visual spelling, visual rhyming, and auditory rhyming judgment in order to examine brain activation for different aspects of language/reading processing. There might be a greater involvement of orthographic processing in the visual spelling and visual rhyming tasks than the auditory rhyming task, and there might be a greater involvement of phonological processing in the two rhyming judgment tasks than the visual spelling task. We aimed to evaluate brain differences that are persistent across development, more evident in children than in adults with RD and more evident in adults than in children with RD using multiple tasks.

## 2 Methods

### 2.1 Participants

We recruited 83 fifth-grade children from public elementary schools and 94 college students from associate degree schools in the local areas. The inclusion criteria for individuals with RD were as follows: (1) a standard score on the Raven nonverbal IQ test (Raven et al., 2004) above 80 and (2) a z score against the normed sample below -1 on at least one of three tests, namely, a character naming test, a sentence reading fluency test, and a 1-minute character naming test. Children with RD and adults with RD had matched socioeconomic status (SES) in terms of years of maternal education. The inclusion criteria for ACs were as follows: (1) an age matched with that of the participants in the corresponding RD group (*t*(81) = 1.67,*p*= .100 for the children;*t*(92) = 0.76,*p*= .778 for the adults), (2) a standard score on the Raven test above 80, and (3) a z score against the normed sample above -1 on all three tests. The sample included 42 typical children (age range: 10–12 years), 41 children with RD (age range: 10–12 years), 49 typical adults (age range: 19–25 years), and 45 adults with RD (age range: 19–26 years) (Table 1). Typical participants and participants with RD were recruited from the same schools. All participants were native Chinese speakers, right-handed (Oldfield, 1971), free of neurological disease or psychiatric disorders, and did not have attention deficit hyperactivity disorder, autism, or stuttering. None of our participants has had reading interventions of any kind.

**Table 1:** Demographic information and behavioral test scores for the four groups of participants.

	Children		Adults	
	AC	RD	AC	RD
N	42 (14 M)	41 (30 M)	49 (14 M)	45 (21 M)
Age (Year)	11.23 (0.54)	11.05 (0.43)	20.73 (1.59)	20.64 (1.28)
Maternal education (Year)		9.20 (2.94)		8.29 (2.40)
Raven	106.02 (9.00)	103.78 (5.89)	118.76 (11.15)	114.40 (11.99)
Character naming (150)	131.10 (7.14)	87.63 (18.65) ^***^	143.20 (2.92)	136.64 (7.84) ^*^
Sentence reading fluency (3505)	1173.36 (284.68)	542.83 (180.20) ^***^	1777.29 (357.75)	845.51 (340.41) ^***^
1-minute character naming (150)	68.74 (15.21)	32.16 (14.58) ^***^	98.08 (14.23)	57.68 (13.07) ^***^
Character dictation (40)	27.79 (5.72)	15.71 (7.10) ^***^	37.50 (2.94)	32.28 (7.32) ^*^
Initial sound deletion (30)	20.88 (7.02)	11.16 (7.93) ^***^	25.69 (3.51)	16.18 (9.96) ^***^
Pseudoword rhyming (40)	31.95 (3.98)	28.27 (4.30) ^***^	36.00 (2.50)	32.38 (3.10) ^***^
Delayed copy (30)	22.88 (3.37)	19.41 (2.87) ^***^	27.14 (1.86)	25.31 (2.58) ^**^
Orthographic correction (60)	41.01 (8.76)	30.39 (5.09) ^***^	49.49 (4.31)	45.10 (6.65) ^**^
Homographic morpheme (30)	24.12 (2.80)	17.68 (3.33) ^***^	26.80 (2.22)	25.69 (2.77)
Homophonic morpheme (30)	27.76 (2.02)	21.49 (4.72) ^***^	29.67 (0.69)	28.89 (1.13)
Digit span	7.55 (1.41)	7.46 (1.36)	9.82 (0.99)	9.60 (1.36)
Picture memory	2.28 (0.90)	2.34 (0.80)	5.49 (1.89)	5.21 (1.78)
Digit RAN	29.67 (5.79)	43.03 (9.64) ^***^	23.60 (3.54)	28.56 (4.41) ^***^
Picture RAN	45.74 (6.31)	67.39 (18.14) ^***^	40.80 (6.41)	50.86 (9.58) ^***^
Paragraph listening comprehension (9)	5.95 (1.89)	5.10 (1.66) ^**^	7.73 (0.84)	6.44 (1.22) ^**^

* , significant differences between RD and AC.^*^p< .05;^**^p< .01;^***^p< .001. The maximum score of the test is indicated by the number in the parentheses following the test name. For the digit span task and the picture memory task, the score is the maximum number of items that participants can remember. For the digit RAN task and the picture RAN test, the score is the mean time needed by participants to complete the test in seconds.

AC, age-matched controls; RD, individuals with reading disability.

The Institutional Review Board in the Sun Yat-sen University approved the consent procedures. Written consent from adult participants and parent consent and child assent were obtained before the experiment. Only some of the participants in each group participated in the fMRI experiments (Table S1). Most of the adults with RD participated in all three fMRI tasks, while in the other three groups, people who participated in the visual rhyming task were different than those who participated in the auditory rhyming and visual spelling tasks, and participants in the latter two tasks were mostly the same. This was because participants did not want to come back for a second scan after we scanned for the auditory rhyming and the visual spelling tasks, and we recruited new participants for the visual rhyming task. One behavioral session and two fMRI sessions were included in this study.

### 2.2 Behavioral tests

#### 2.2.1 Reading and writing tests

Three tests, namely, the character naming, reading fluency, and 1-minute character naming tests, were used to measure reading performance. In the character naming test, the participants were instructed to read aloud each character without a time limit. In the sentence reading fluency test, the participants were given 3 minutes to silently read each sentence and instructed to judge whether each sentence made sense in terms of meaning. The character naming and sentence reading fluency tests included 150 characters and 100 sentences, respectively. The reliability of both tests as measurements of Chinese literacy has been previously confirmed (Song et al., 2015;Xue et al., 2013). To establish a standard against which to compare the fifth-grade children who participated in our study, previously reported test results of 264 typical fifth graders were used (Song et al., 2015). Similarly, results from 203 typical college students from associate degree schools who participated in the character naming test and 215 typical college students who participated in the sentence reading fluency test were used as standards for these two tests for the adult participants (mean age: 19.83 years; mean score: 140.02, standard deviation: 6.41 for the character naming test; mean age: 19.83 years; mean score: 1379.88, standard deviation: 377.21 for the sentence reading fluency test).

For the 1-minute character naming test, the participants were asked to read as many single characters out loud as possible within 1 minute. Both regular and irregular characters were included. Regular characters included those that share the same phonology as the phonetic radical, such as 铭 (ming2), in which the phonetic radical 名 (ming2) has the same phonology as the character. Irregular characters included those that have different phonology from the phonetic radical, such as 错 (cuo4), in which the phonetic radical 昔 (xi1) has a different phonology from the character. We further tested 217 typical fifth-grade children and 201 typical college students, and the mean results were used as norms for this test (mean score: 71.18, standard deviation: 18.11 for regular characters in children; mean score: 48.72, standard deviation: 18.29 for irregular characters in children; mean score: 100.45, standard deviation: 17.70 for regular characters in adults; and mean score: 80.91, standard deviation: 19.10 for irregular characters in adults).

A character dictation test was used to measure spelling. During the test, participants listened to two-character words pronounced orally and were instructed to write down one of the characters from each word. The test comprised a list of 40 words.

#### 2.2.2 Cognitive-linguistic tests

To measure phonological awareness, we used an initial sound deletion test and a pseudoword rhyming judgment task. English words and pseudowords were used as stimuli for the two tests, because Chinese characters are all monosyllabic which may cause a ceiling effect in adult participants. Using pseudowords also avoided different stimulus familiarity in adults versus children and in typical readers versus RD readers. To measure orthographic skills, we used a delayed copy test and an orthographic correction test. In the delayed copy test, pseudocharacters were presented for 500 ms, and the participants were asked to write down the pseudocharacter they had just seen. Pseudocharacters were generated by switching the position of radicals in real characters. In the orthographic correction test, the participants were asked to correct a miswritten character by adding, deleting, or changing the strokes. Regarding morphological tests, we used a homophonic morpheme test, in which the participants were asked to choose the correct character for a target word from a few homophones, and a homographic morpheme test, in which the participants were asked to choose the correct meaning of the morpheme in a target word. To evaluate short-term memory, we used digit span and picture memory tests. Furthermore, we used a digit rapid automatized naming (RAN) test and a picture RAN test to evaluate rapid naming skills. The time that the participants needed to name the whole list was measured. We tested listening comprehension using a paragraph listening comprehension test. The participants were asked to answer 9 multiple-choice questions about the paragraph they had just heard.

### 2.3 fMRI tasks

An auditory rhyming, a visual rhyming, and a visual spelling task were used in the fMRI scanner, with the auditory rhyming and the visual spelling tasks administered during one visit and the visual rhyming task administered during another visit, and the order of the tasks was counterbalanced. Within each task, there were 96 lexical trials, 24 perceptual baseline trials, and 48 null baseline trials. For each lexical trial, there were two sequentially presented visual or auditory two-character words. In the auditory rhyming and the visual rhyming judgment tasks, participants were asked to judge whether the two words rhymed or not, and in the visual spelling task, participants were asked to judge whether the second characters of the two words had similar orthography by sharing a phonetic radical. For the two visual tasks, we used Tibetan symbols as perceptual baselines and for the auditory task, we used pure tones as perceptual baselines. For the perceptual trials, participants needed to judge whether the two Tibetan symbols or pure tones were identical or not. For the null baseline trials, participants were instructed to press the “yes” button when they saw a black cross.

For each trial, each of the two stimuli was presented for 800 ms, with an interval of 200 ms between them. There was a 2200–3400 ms jittered interval between trials. The presentation order of all trials was randomized and optimized using OptSeq (http://surfer.nmr.mgh.harvard.edu/optseq) for each task. More details about the materials and procedures are presented in theSupplementary Materials.

### 2.4 MRI data acquisition

MRI data were acquired using a Siemens 3T Prisma MRI scanner with a standard 20-channel head coil. T1-weighted images were collected using an MP-RAGE sequence with the following parameters: TR = 2300 ms, TE = 3.24 ms, TI = 900 ms, flip angle = 9°, matrix size = 256 × 256, field of view = 260 mm, slice thickness = 1 mm, and number of slices = 160. Functional images were collected using an EPI sequence with the following parameters: TR = 2000 ms, TE = 20 ms, flip angle = 80°, matrix size = 128 × 128, field of view = 220 mm, slice thickness = 3 mm, number of slices = 34, and image resolution = 1.7 × 1.7 × 3.0 mm.

### 2.5 fMRI data analysis

The following analyses were conducted for fMRI data preprocessing using SPM12 (http://fil.ion.ucl.ac.uk/spm): (1) slice timing correction for interleaved acquisition of images using sinc interpolation, (2) 4th-degree b-splice interpolation for realignment of brain volumes to the first volume for each run, (3) trilinear coregistration of functional images with anatomical images for each individual, (4) segmentation of the anatomical images, (5) normalization of all functional images to the Montreal Neurological Institute (MNI) template, with parameters derived from the structural image, and (6) 4 mm full-width half-maximum (FWHM) Gaussian kernel smoothing for the functional data. For motion correction, we first defined outlier time points when the slice-to-slice head movement exceeded 3 mm or 0.05 radian, and then we counted the number of outlier time points for each participant in each run using the Art toolbox (https://www.nitrc.org/projects/artifact_detect/). If outlier time points accounted for 10% or more of the total time points, the participant was removed from further analysis; otherwise, the outlier time points were scrubbed. Using this criterion, only one adult with RD in the visual spelling task was removed from further analysis.

After the data were preprocessed, a general linear model (GLM) with a high-pass filter of 128 seconds and six head movement parameters included as nuisance regressors was calculated for individual analysis. The contrast of the lexical minus null trials was used for further group-level analyses. Group (ACs, RD) by age (adults, children) ANOVAs were used to reveal the main effects of group and age, as well as the interaction between group and age, for each task. All significant results were reported at the threshold of*p*< .005 uncorrected at the voxel level and FDR-corrected*p*< .05 at the cluster level. To determine the common RD effects across tasks, conjunction analyses across tasks were conducted (Friston et al., 1999;Nichols et al., 2005) separately for children and adults. The conjunction results were reported using xjView (https://www.alivelearn.net/xjview/), and only clusters with a size exceeding 10 voxels were reported. For regions that showed common RD effects across all tasks in children but not in adults, or vice versa, we conducted VOI analyses to examine whether the RD effects were significantly different in different ages, tasks, and brain regions. A sphere with a radius of 6 mm centered at the peak coordinate was created, and the beta value within the sphere was extracted for each participant in each task and used in a group (ACs, RDs) by age (children, adults), by task (auditory rhyming, visual rhyming, visual spelling), and by brain regions (IFG, ITG) ANCOVA, with accuracy in each task as a covariate in the VOI analysis.

To examine the relationship between brain activity and behavioral differences, we correlated brain activation in regions with common deficits across all tasks with the behavioral test for which the most significant deficit was observed separately for children and adults with RD using Pearson’s correlation. In particular, we correlated brain activation in the left ITG with the score on the sentence reading fluency test for adults with RD, and we correlated brain activation in the left IFG with the score on the character naming test for children with RD.

### 2.6 Verification analyses: Psychophysiological interaction (PPI) analysis

To understand why some brain differences are more severe in children with RD and some are more severe in adults with RD, we examined functional connectivity between the regions of interest from the brain activation analysis and the rest of the brain using psychophysiological interaction (PPI) analyses. Two VOIs were identified as seed regions based on the brain activation analysis results (i.e., the left IFG and the left ITG), and individualized VOIs were generated by finding the activation peak for each individual in each VOI. Then, time series were extracted from a 6 mm-radius sphere centered at the individualized peak for each participant for each seed. After the first-level PPI analysis, group (ACs, RD) by age (Adults, Children) ANCOVAs were conducted separately for each seed and each task with accuracy of the task as a covariate. All significant results were reported at the threshold of*p*< .005 uncorrected at the voxel level and FDR-corrected*p*< .05 at the cluster level. For regions that showed a significant interaction between group and age in the PPI analysis, connection values were extracted for further simple effect analysis.

## 3 Results

### 3.1 Results on the behavioral tests

**Fig. 1.**
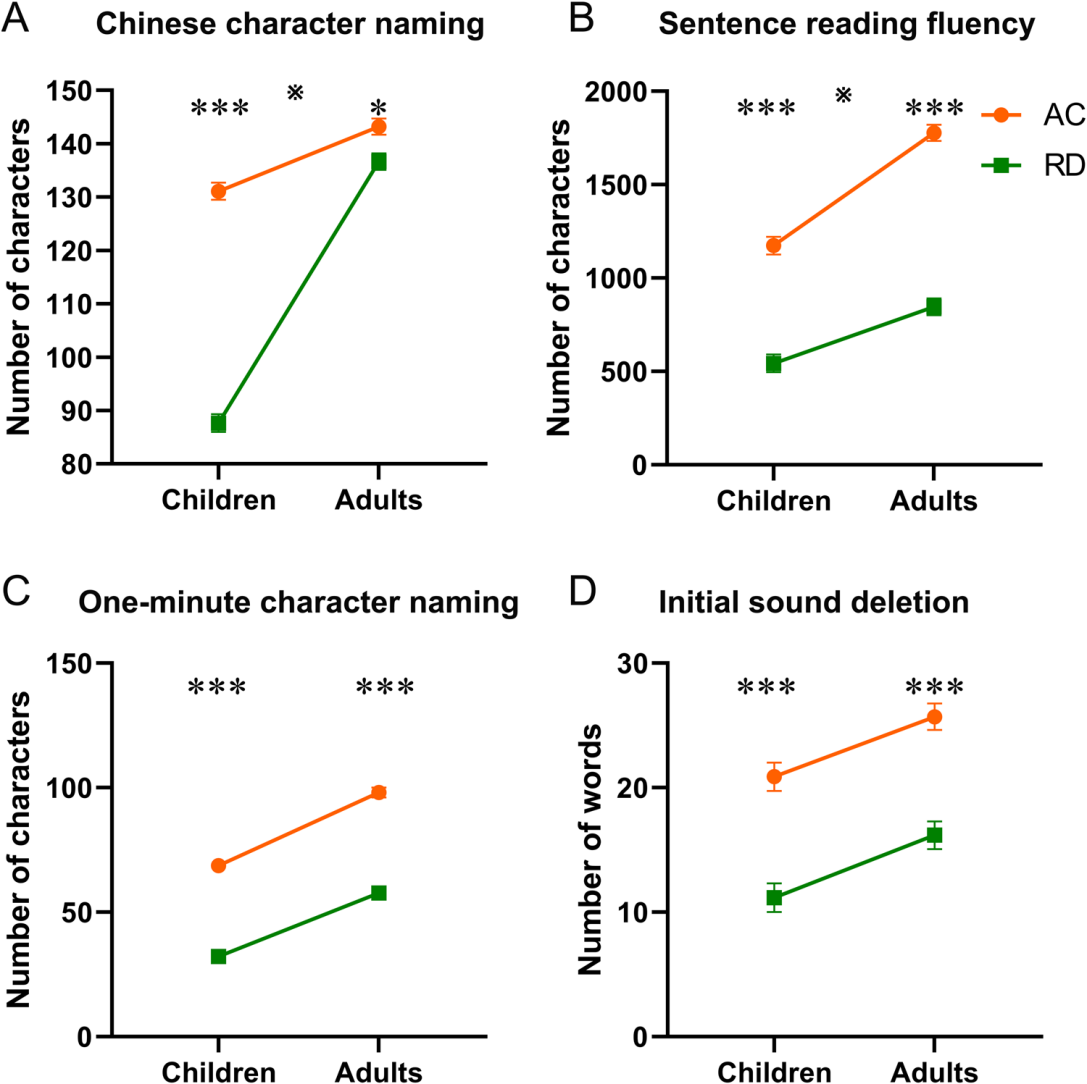
Age-related changes in some behavioral tests for typical readers and RD readers. (A) Greater deficits were found in children than adults with RD in the character naming test. (B) Greater deficits were found in adults than children with RD in the sentence reading fluency test. (C, D) Similar amount of deficits was found in the 1-minute character naming and the initial sound deletion tests. ※ indicates a significant interaction between age and group in the ANOVA for each task.^*^p< .05;^***^p< .001.

For each behavioral test (Table 1;Fig. 1), we ran a group by age ANOVA. There was a significant interaction between age and group for the character naming test (Fig. 1A;*F*(1, 173) = 135.65,*p*< .001,*η^2^*= .150). Simple effect analysis revealed that for the character naming test, children with RD showed greater deficits (*F*(1, 174) = 111.16,*p*< .001,*η^2^*= .384) than adults with RD (*F*(1, 174) = 4.10,*p*= .044,*η^2^*= .014). Alternatively, this could be interpreted as a greater developmental increase in RD readers than in typical readers (*F*(1, 174) = 16.53,*p*< .001,*η^2^*= .044 for typical readers;*F*(1, 174) = 183.10,*p*< .001,*η^2^*= .490 for RD readers). For the character dictation, orthographic correction, delayed copy, homographic morpheme, homophonic morpheme, digit rapid automatized naming (RAN), and picture RAN tests, there was also a significant interaction between age and group, which was driven by greater deficits in children with RD than in adults with RD (seeTable 1for details).

For the sentence reading fluency test, there was also a significant interaction (Fig. 1B;*F*(1, 173) = 10.90,*p *= .001,*η^2^*= .018), driven by greater deficits in adults with RD (*F*(1, 174) = 147.95,*p*< .001,*η*^2^= .391) than children with RD (*F*(1, 174) = 56.56,*p*< .001,*η^2^*= .149). Alternatively, this could be interpreted as a greater developmental increase in typical readers than in RD readers (*F*(1, 174) = 40.52,*p*< .001,*η^2^*= .184 for typical readers;*F*(1, 174) = 6.16,*p*= .014,*η^2^*= .028 for RD readers).

For the 1-minute character naming (Fig. 1C), initial sound deletion (Fig. 1D), pseudoword rhyming, and paragraph listening comprehension tests, there were main effects of group and age but no significant interactions between age and group. The main effect of group was due to lower performance in individuals with RD than in ACs, and the main effect of age was due to lower performance in children than in adults. We found no main effects or interaction effects for the digit span or picture memory tests.

### 3.2 Behavioral performance on the in-scanner tasks

The detailed behavioral results from ANOVAs of group by age by task on accuracy and reaction times are reported in the Supplementary Materials (Fig. S1). For accuracy, we found a significant interaction of group by task, driven by lower accuracy in RD readers than AC readers for the auditory rhyming task and the visual rhyming task, but not the visual spelling task. We found a significant interaction of age by task, with a greater age-related increase in accuracy for the auditory rhyming task than the visual rhyming and visual spelling tasks. We found a significant interaction of age by group, driven by a greater decrease of accuracy in children with RD compared to ACs than in adults with RD.

For reaction time, we found a significant interaction of age by group by task, driven by slower reaction time in adults with RD than ACs but not in children with RD in the auditory rhyming task, and slower reaction time in children with RD than ACs but not in adults with RD in the visual spelling task.

### 3.3 Brain activation results

#### 3.3.1 Differences between RD readers and typical readers

The main effect of group in the ANOVA of group (RD, ACs) by age (children, adults) for each task is reported in the Supplementary Materials (Table S2;Fig. S2).

**Fig. 2.**
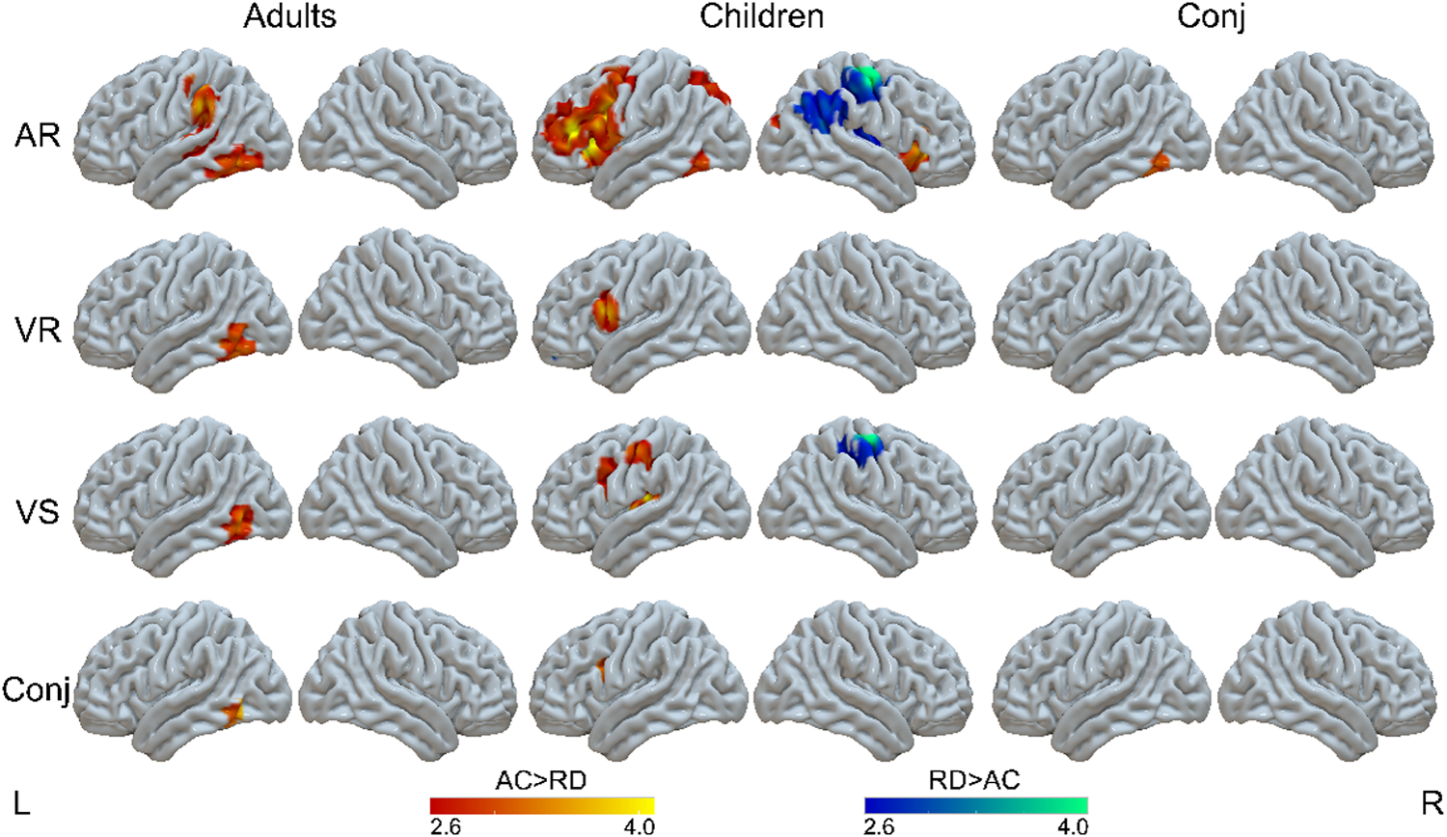
Differences between ACs and RD readers for each task separately for adults and children (*p *< .005 uncorrected at the voxel level, FDR corrected*p*< .05 at the cluster level). AR, auditory rhyming; VR, visual rhyming; VS, visual spelling; Conj, conjunction of the group differences across all tasks for children or adults or conjunction of group differences across adults and children for each task.

Furthermore, in order to examine common RD effects across tasks within each age group, we analyzed group effects for children and adults separately in each task and then ran a conjunction analysis. The regions with a significant group difference separately for children and adults in each task are shown inFigure 2and listed inTable S3.**A conjunction analysis showed an overlap of the RD effects (i.e., greater activation in ACs than RD) across tasks in the left ITG, peaking at -50, -56, -14, with a cluster of 69 voxels for adults with RD**(Table 2;Fig. 2). For the contrast of RD>ACs, adults with RD did not show greater activation than ACs in any part of the brain.

**Table 2:** RD effects, age-related changes, and their interaction in the brain.

Brain regions	H	BA	Voxels	MNI coordinate			z
**Common RD effects across children and adults in each task (ACs>RD)**							
**Auditory rhyming**							
Inferior temporal gyrus	L	19/37	106	-48	-58	-16	-
**Visual rhyming**							
-	-	-	-	-	-	-	-
**Visual spelling**							
-	-	-	-	-	-	-	-
**Common RD effects across children and adults in each task (RD>ACs)**							
-	-	-	-	-	-	-	-
**Common RD effects across tasks in children (ACs>RD)**							
Inferior frontal gyrus	L		15	-42	8	26	-
**Common RD effects across tasks in children (RD>ACs)**							
-	-	-	-	-	-	-	-
**Common RD effects across tasks in adults (ACs>RD)**							
Inferior temporal gyrus	L	19	69	-50	-56	-14	-
**Common RD effects across tasks in adults (RD>ACs)**							
-	-	-	-	-	-	-	-
**Common age-related increases across ACs and RD in each task**							
**Auditory rhyming**							
Inferior frontal gyrus	L	46	132	-44	26	18	-
Superior temporal gyrus	L	22	106	-60	-18	6	-
Inferior temporal gyrus	L	37	115	-50	-50	-14	-
Medial frontal gyrus	R		13	12	0	50	-
Precentral gyrus	R	6/9	63	54	0	40	-
Precentral gyrus	R	6	48	34	-14	54	-
Superior temporal gyrus	R	22	241	66	-16	4	-
Superior temporal gyrus	R	38	33	52	6	-14	-
**Visual rhyming**							
Inferior frontal gyrus	L	46	250	-44	28	18	-
Inferior frontal gyrus	L	9	189	-50	12	30	-
Inferior temporal gyrus	L	37	257	-52	-50	-12	-
Inferior occipital gyrus	L	18	173	-34	-86	-14	-
Inferior occipital gyrus	R	18/19	101	38	-86	-10	-
**Visual spelling**							
Inferior frontal gyrus	L	9	26	-50	14	28	-
Superior parietal lobule	L	7	102	-18	-70	56	-
Inferior occipital gyrus	L	37	227	-46	-72	-14	-
Middle occipital gyrus	L	19	34	-46	-76	0	-
Lingual gyrus	R	17	202	16	-84	-6	-
Middle occipital gyrus	R	19	146	34	-81	12	-
**Common age-related decreases across ACs and RD in each task**							
**Auditory rhyming**							
Inferior occipital gyrus	L	18/19	158	-40	-80	-10	-
Medial frontal gyrus	R	9	19	4	38	34	-
Middle frontal gyrus	R	8/9	220	38	32	38	-
Lingual gyrus	R	18	98	26	-84	-10	-
**Visual rhyming**							
-	-	-	-	-	-	-	-
**Visual spelling**							
Middle frontal gyrus	R		37	38	22	40	-
**Common age-related increases in ACs across tasks**							
Inferior temporal gyrus	L	37	171	-50	-50	-14	-
**Common age-related decreases in ACs across tasks**							
-	-	-	-	-	-	-	-
**Common age-related increases in RD across tasks**							
Inferior frontal gyrus	L	46	276	-48	26	20	-
Inferior frontal gyrus	L	6	21	-48	2	46	-
Inferior frontal gyrus	L	9	205	-46	10	24	-
Inferior parietal lobule	L	7	147	-28	-60	38	-
Inferior temporal gyrus	L	20	24	-50	-50	-16	-
**Common age-related decreases in RD across tasks**							
Supramarginal gyrus	R	40	183	60	-30	38	-
**Common age-related increases across tasks and across ACs and RD**							
Inferior temporal gyrus	L	20	23	-50	-50	-14	-
**Interaction**							
**Auditory rhyming**							
Supplementary motor area	L	6/8/32	399	-2	16	48	4.91
Putamen	L		535	-16	4	8	4.64
Inferior frontal gyrus	L	46	234	-42	38	14	4.37
Insula	L	13/47	218	-32	22	4	4.26
Insula	R	13/47	199	32	20	-8	4.21
Cerebellum	R		356	6	-74	-30	4.09
Inferior frontal gyrus	L	9/44/45	231	-58	16	28	3.93
Medial frontal gyrus	L	6	139	-20	2	58	3.90
Middle frontal gyrus	L	10	179	-30	56	16	3.80
Cerebellum	R		170	28	-64	-30	3.70
Precentral gyrus	R	3/4/6	852	32	-22	52	6.14
Precuneus		7/31	1676	-12	-52	30	5.01
Insula	R		126	46	0	12	4.62
Hippocampus	L		165	-24	-8	-18	4.15
Putamen	R		153	32	-14	2	4.00
Superior temporal gyrus	R		159	56	-8	-4	3.99
Supramarginal gyrus	R	39/40	381	46	-52	24	3.97
Superior temporal gyrus	L	41	123	-46	-36	12	3.59
Medial frontal gyrus	L	10	204	-10	46	-6	3.52
**Visual rhyming**							
Putamen	L		241	-16	8	2	3.91
**Visual spelling**							
Thalamus	L		316	-12	-10	4	3.84
Precentral gyrus	R	3/4	382	38	-20	58	5.21

For children with RD,**the conjunction analysis showed an overlap of the RD effects (i.e., greater activation in ACs than RD) across all tasks in the left IFG, peaking at -42, 8, 26, with a cluster of 15 voxels**(Table 2;Fig. 2).

For the contrast of RD>ACs, children with RD showed greater activation than ACs in each task, but the conjunction analysis did not reveal common effects (Table 2;Fig. 2).

The conjunction analysis of RD effects across children and adults for each task showed common RD effects in the left ITG for the auditory rhyming task. When we lowered the threshold to*p*< .005 uncorrected at the voxel level and >20 voxels in the cluster level, we found an additional overlap between children and adults in the left IPL (-48, -42, 34) for reduced brain activation in individuals with RD than controls (Fig. S3). No common RD effects in children and adults were observed for the visual rhyming and visual spelling tasks (Table 2;Fig. 2).

#### 3.3.2 Differences between children and adults

**Fig. 3.**
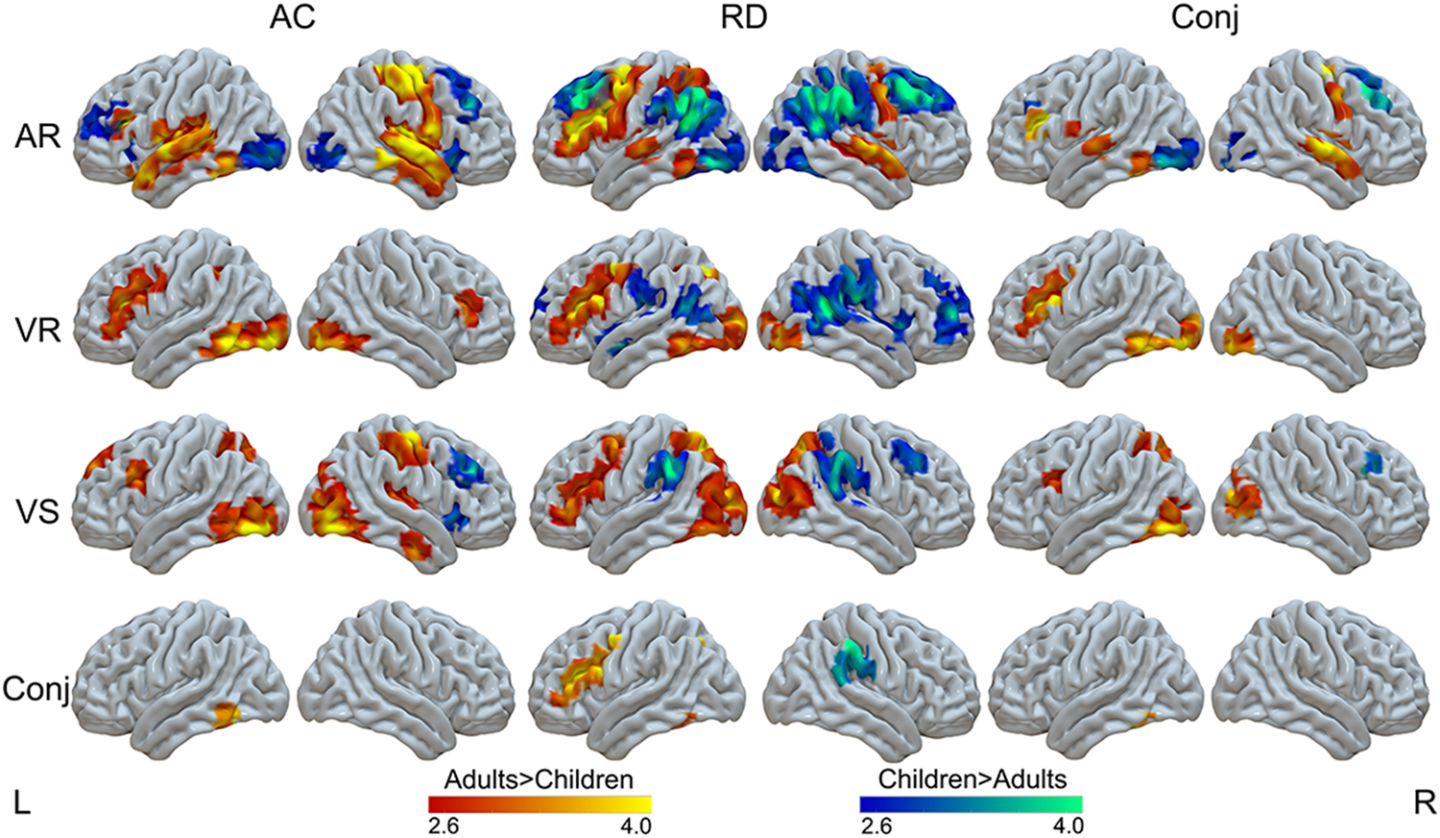
Age-related changes in ACs and RD readers for each task (*p *< .005 uncorrected at the voxel level, FDR corrected*p*< .05 at the cluster level). The conjunction analysis across AC and RD readers for each task, as well as the conjunction across all tasks for AC and RD readers separately are also presented.

The main effects of age in the ANOVA of group by age for each task are reported in the Supplementary Materials (Table S2;Fig. S2). Furthermore, because we expected that the age effects might be different in typical readers and RD readers, we analyzed the two groups separately. The developmental changes in typical readers and RD readers for each task are reported inTable S4andFigure 3. The conjunction analysis showed a common developmental increase in the left ITG in typical readers across all three tasks. However, this analysis did not show a common developmental decrease in typical readers. Moreover, it showed several common developmental increases in RD readers across tasks, including three clusters in the left IFG, one cluster in the left inferior parietal lobule, and one cluster in the left ITG. Furthermore, the conjunction analysis showed a common developmental decrease in the right supramarginal gyrus in RD readers across tasks (Table 2;Fig. 3).

We also looked at the conjunction of age effects across typical and RD readers for each individual task. Detailed reports are in theSupplementary Materials. Finally, a conjunction of age-related increase across all tasks and all participants was found in the left ITG (Table 2;Fig. 3).

#### 3.3.3 Interaction effects

**Fig. 4.**
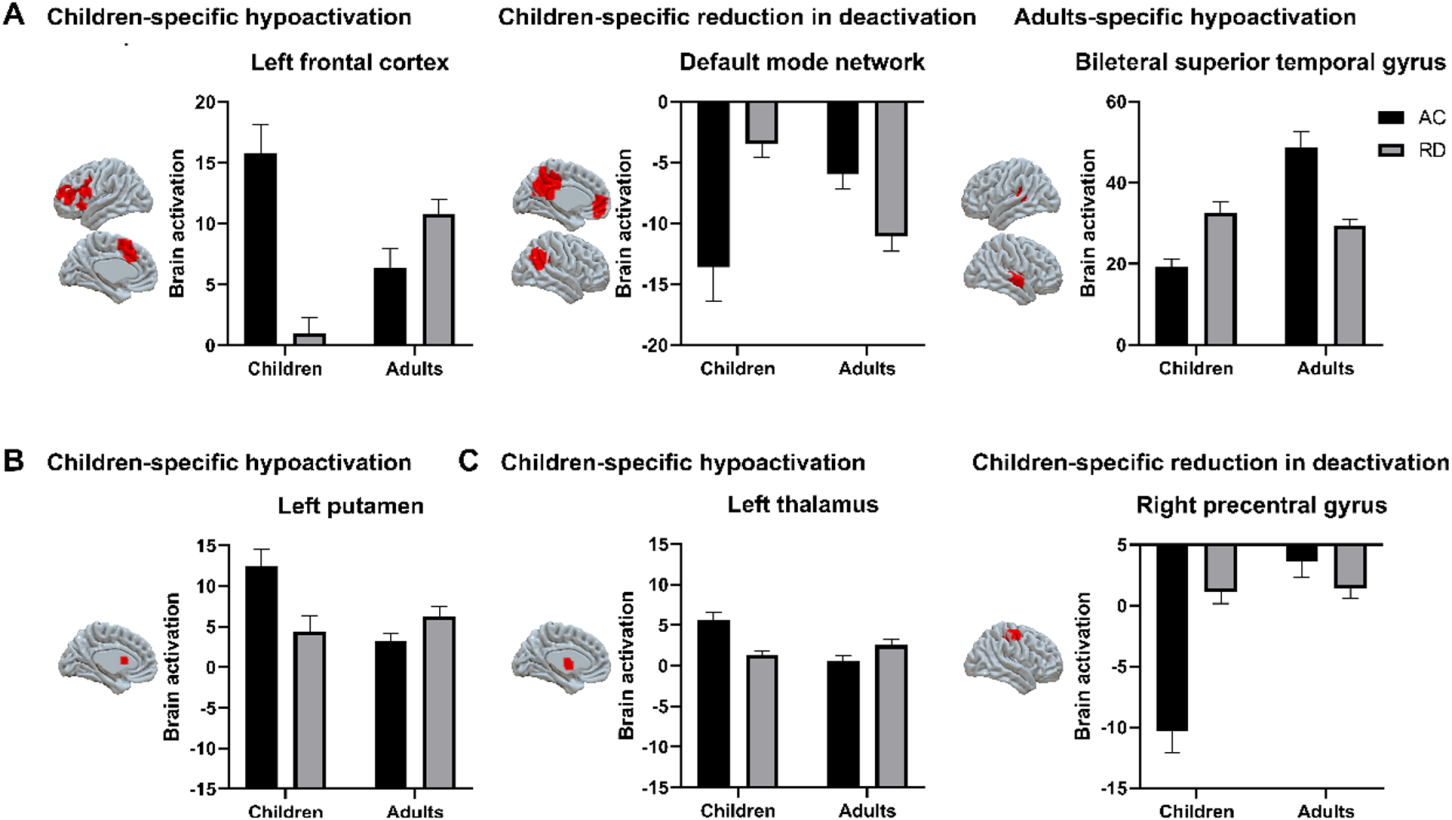
The interaction effects at the whole-brain level between group and age for each task. (A) The interaction for the auditory rhyming task. (B) The interaction for the visual rhyming task. (C) The interaction for the visual spelling task. (Note: seeFig. S4for all regions that showed interactions.)

The brain regions with a significant interaction between group and age from the whole-brain ANOVA are presented inTable 2,Figure 4, andFigure S4. Further simple effect analysis showed that for the auditory rhyming task, the interaction was driven by reduced activation in children but not in adults with RD in regions of the left frontal cortex (i.e., the left IFG, left insula, left middle frontal gyrus, and left medial frontal gyrus), as well as at the left putamen, right insula, and right cerebellum. The interaction was driven by reduced deactivation in children but not in adults with RD in the default mode network (i.e., the left medial frontal gyrus, left precuneus, right putamen, right insula, right precentral gyrus, and right supramarginal gyrus). Furthermore, the interaction was driven by reduced activation in adults but not in children with RD in the bilateral superior temporal gyri and left hippocampus.

The interaction for the visual rhyming task was driven by reduced activation in children but not adults with RD in the left putamen. The interaction in the visual spelling task was driven by reduced activation in children but not in adults with RD in the left thalamus and reduced deactivation in children but not in adults with RD in the right precentral gyrus.

#### 3.3.4 VOI results

**Fig. 5.**
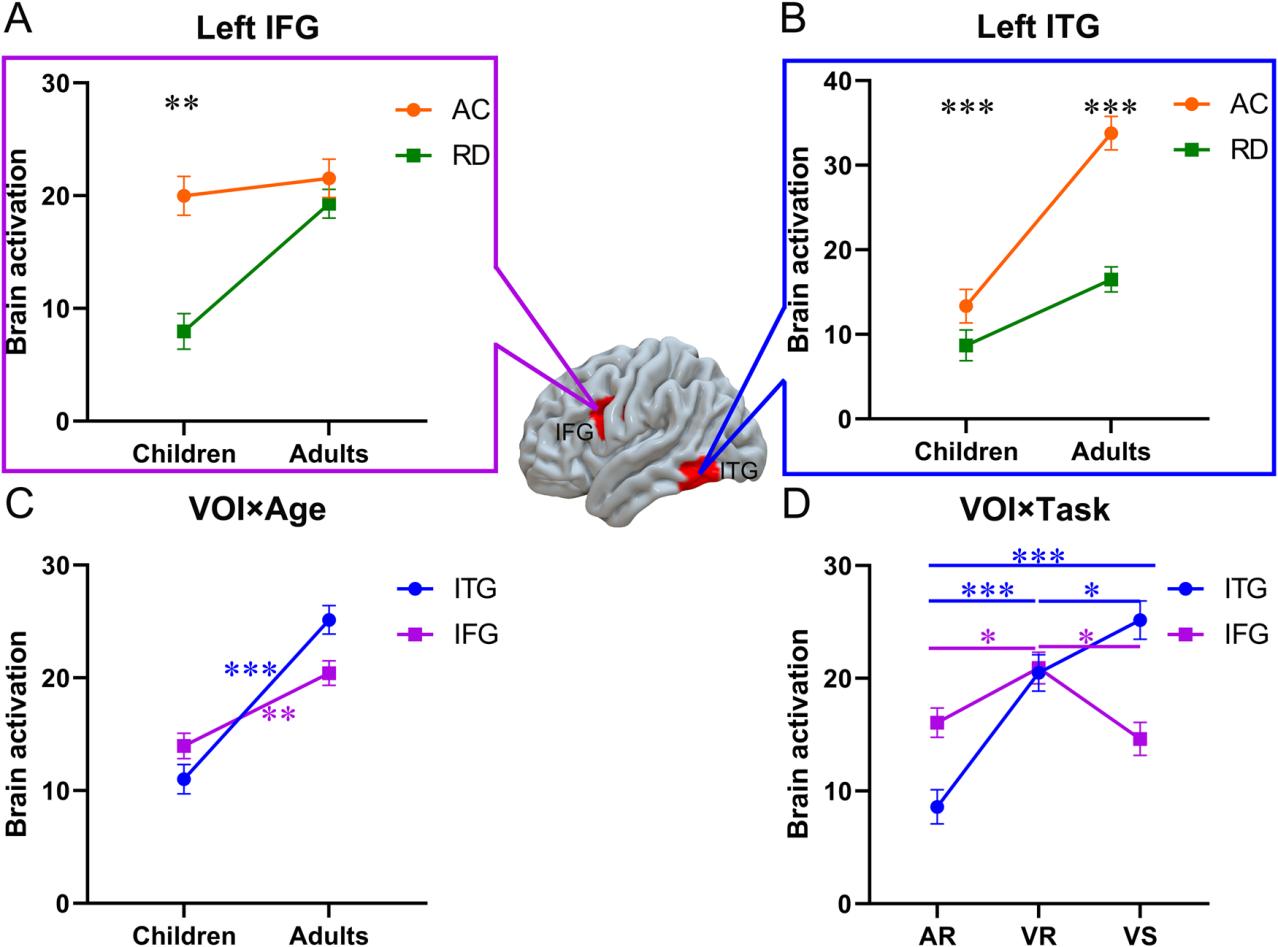
Significant interactions in the VOI analysis. (A, B) Significant interaction of group × age × VOI driven by greater reduction of brain activation in children than adults with RD in the left IFG and greater reduction in adults than children with RD in the left ITG. (C) Significant interaction of VOI × age driven by greater age-related increase in the left ITG than the left IFG. (D) Significant interaction of VOI × task, driven by greater activation in the visual spelling task than the rhyming tasks in the left ITG and greater activation in the rhyming tasks than the visual spelling task in the left IFG. (AR, the auditory rhyming task; VR, the visual rhyming task; VS, the visual spelling task).^*^p< .05;^**^p< .01;^***^p< .001. Blue star indicates significant results in left ITG, and purple star indicates significant results in left IFG.

We identified the left IFG and left ITG as volumes of interest (VOI) due to the persistent activation differences across all tasks observed in children and adults with RD, respectively. Then, we extracted beta values and conducted a group × age × task × VOI ANCOVA with the in-scanner task accuracy of each task as a covariate. We found a significant group × age × VOI interaction (*F*(2, 300) = 37.31,*p*< .001,*η^2^*= .028), driven by reduced brain activation in children but not in adults with RD in the left IFG (*F*(1, 309) = 7.31,*p*= .007,*η^2^*= .012 for children,*F*(1, 309) = 0.88,*p*= .349,*η^2^*= .001 for adults) (Fig. 5A), and greater reduction of brain activation in adults than in children with RD in the left ITG (*F*(1, 309) = 3.02,*p*< .001,*η^2^*= .005 for children,*F*(1, 309) = 13.91,*p*< .001,*η^2^*= .022 for adults) (Fig. 5B). This interaction can also be explained by greater developmental increases in RD readers than in typical readers in the left IFG (*F*(1, 309) = 0.02,*p*= .900,*η^2^*= .001 for AC,*F*(1, 309) = 13.70,*p*< .001,*η^2^*= .022 for RD), and greater developmental increases in typical readers than in RD readers in the left ITG (*F*(1, 309) = 10.91,*p*= .001,*η^2^*= .017 for AC,*F*(1, 309) = 2.52,*p*= .114,*η^2^*= .004 for RD).

We also found a significant age × VOI interaction (*F*(1, 300) = 14.83,*p*< .001,*η^2^*= .011), driven by greater developmental increases in the left ITG than the left IFG (*F*(1, 309) = 47.18,*p*< .001,*η^2^*= .074 for ITG,*F*(1, 309) = 9.81,*p*= .002,*η^2^*= .015 for IFG) (Fig. 5C).

We found a significant task × VOI interaction (*F*(2, 300) = 23.96,*p*< .001,*η^2^*= .036), driven by greater task difference in the left ITG than the left IFG (*F*(1, 309) = 7.87,*p*< .001,*η^2^*= .025 for left ITG,*F*(1, 309) = 3.42,*p*= .034,*η^2^*= .011 for left IFG). Further post-hoc analyses showed that brain activation in the left IFG during the visual rhyming task was greater than that during the visual spelling task (*t*(309) = 2.78,*p*= .006,*FDRp*= 0.018) and the auditory rhyming task (*t*(309) = 2.39,*p*= .017,*FDRp*= 0.026); brain activation in the left ITG during the visual spelling task was greater than that during the auditory rhyming task (*t*(309) = 7.20,*p*< .001,*FDRp *< 0.001) and the visual rhyming task (*t*(309) = 2.07,*p*= .039,*FDRp*= 0.039); and brain activation in the left ITG during the visual rhyming task was greater than that during the auditory rhyming task (*t*(309) = 5.86,*p*< .001,*FDRp *< 0.001) (Fig. 5D). Other results were reported inSupplementary Materials.

### 3.4 Brain behavioral correlations

**Fig. 6.**
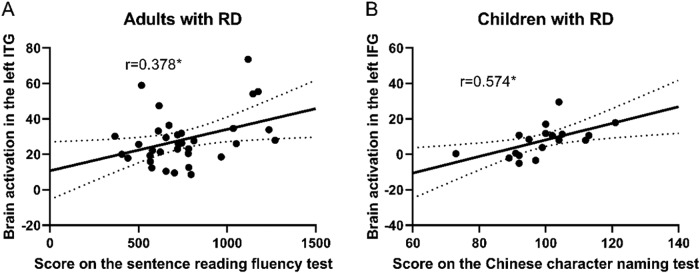
Brain-behavior correlations in the left ITG and left IFG. (A) Brain activation in the left ITG during the visual spelling task was positively correlated with scores on the sentence reading fluency test in adults with RD. (B) Brain activation in the left IFG during the visual rhyming task was positively correlated with scores on the Chinese character naming test in children with RD.^*^p< .05.

To understand the brain function of the left IFG and left ITG, we performed brain behavioral correlation analyses, which showed that brain activation in the left ITG during the visual spelling task was positively correlated with sentence reading fluency scores in adults with RD (*r*= 0.378,*p*= .027,*FDR_p_*= 0.080). A significant positive correlation was also found between brain activation in the left IFG during the visual rhyming task and the score in the character naming test in children with RD (*r*= 0.574,*p*= .013,*FDR_p_*= 0.039) (Fig. 6).

### 3.5 Verification analyses: Psychophysiological interaction (PPI) results

To understand why different abnormal patterns occurred in children and adults with RD, we performed psychophysiological interaction (PPI) analyses with the left IFG and left ITG as seed regions. PPI results showed that children with RD had reduced connections with the left IFG but adults with RD had normal or increased connections with the left IFG, and the opposite pattern for the left ITG (Fig. S5). We report detailed results in theSupplementary Materials.

## 4 Discussion

In the current study, by directly comparing children and adults with and without RD using both behavioral and neuroimaging measures, we were able to differentiate persistent differences, greater differences in children with RD, and greater differences in adults with RD. Findings also indicate different developmental patterns in typical readers and RD readers.

### 4.1 Persistent deficits

We found persistent deficits in phonological awareness as measured in the initial sound deletion test and word decoding speed, as measured in the 1-minute character naming test. Children and adults with RD showed comparable impairments on these tests, suggesting that phonological deficits and slow reading speed are definitive features of RD which do not disappear with learning and experience. This finding on Chinese individuals with RD is consistent with previous findings that phonological awareness is a persistent deficit in individuals with RD in alphabetic languages (Smith-Spark et al., 2017).

Moreover, we found persistent reduction of brain activation at the left IPL in children and adults with RD in the auditory rhyming task, presumably related with the persistent deficits in phonological awareness. The left IPL has been found to be essential in phonological processing, especially in phonological representation (Vandermosten et al., 2020) and phonological memory (Deschamps et al., 2014), which has demonstrated consistent functional differences even before the onset of reading in children with a high risk of RD in a previous meta-analysis (Vandermosten et al., 2016). A recent meta-analysis found consistent structural and functional differences in the left IPL in individuals with RD across languages (Yan et al., 2021). Taken together, our study adds to the literature that the functional difference in the left IPL may be a neural signature of RD, since it is persistent across ages and languages.

### 4.2 Greater differences in children than adults with RD

We found greater deficits in children than adults with RD on several behavioral tests, including the character naming test, suggesting that these deficits can be overcome to some degree through practice and learning over time. In the brain, for the first time, our fMRI data (whole-brain interaction, and VOI analyses) clearly showed that the functional differences in the left dorsal IFG were only evident in children with RD but disappeared in adults with RD, suggesting delayed development. RD readers had a greater developmental increase in the activation of the left IFG than typical readers across tasks, which is consistent with a previous study in English speakers (Shaywitz et al., 2007). The developmental difference in the left IFG is also consistent with a previous study which found that the left IFG is sensitive to task performance level rather than RD per se (Cao et al., 2020). It lines up with our finding that when the task performance was improved in adults with RD, the brain activation in the left IFG became normal, even though they were RD readers.

It is important to point out that the reason why adults with RD catch up on the left IFG function should not only be due to passive development, but also be due to the experience of active learning and practice. Intervention studies have demonstrated that intensive instruction effectively modulates brain functions in the reading network, including the left IFG (Perdue et al., 2022). Our PPI verification analysis also suggested that the improved brain activation in the left IFG in adults with RD might occur through the development of connections with other parts of the brain, especially in the right hemisphere. This is consistent with other studies suggesting that strong connections with other brain areas may help normalize the brain function in the left IFG (Horowitz-Kraus et al., 2019;Richards & Berninger, 2008;Richards et al., 2018). Reading intervention studies have sounded evidence that the right hemisphere regions are involved to a greater degree following intervention in RD readers than TD readers as a compensatory mechanism (D’Mello & Gabrieli, 2018;Nugiel et al., 2019;Partanen et al., 2019;Perdue et al., 2022;Ullman et al., 2020;Yu et al., 2018). Taken together, continuous learning and practice help RD readers catch up on the function at the left IFG by adulthood, probably by connecting with other brain areas, especially in the right hemisphere as a compensation.

Furthermore, brain-behavioral correlation analysis showed that the left IFG was correlated with word decoding accuracy, as measured in the character naming test in children with RD, suggesting that the left IFG might be related with phonological decoding. The VOI analysis also showed that the left IFG was more involved in the rhyming tasks than the visual spelling task, suggesting its role in phonological decoding. The left IFG has been repeatedly found to be involved in phonological decoding in previous studies (MacSweeney et al., 2008;McDermott et al., 2003). Moreover, the left dorsal IFG has been found to be more involved in Chinese reading than in alphabetic reading (Bolger et al., 2005;Tan et al., 2005), presumably due to the addressed phonological procedures in the Chinese writing system. Greater involvement of the left dorsal IFG in Chinese reading than in alphabetic reading may also be due to greater demand on lexical selection and cognitive control in Chinese because of the existence of multiple homophones (Chen et al., 2009). The left dorsal IFG was found to be more activated for phonologically inconsistent words than consistent words in English (Bolger, Hornickel, et al., 2008;Bolger, Minas, et al., 2008), suggesting its role in lexical selection and cognitive control. A recent meta-analysis study also found that the left dorsal IFG is more impaired in Chinese individuals with RD than in readers of alphabetic languages with RD (Yan et al., 2021). Therefore, Chinese RD is characterized by a failure in engaging the optimal phonological decoding region for their language.

For alphabetic languages, on the other hand, there might be greater difference in children than adults with RD in their phonological decoding area which is thought to be the left temporoparietal area (Paulesu et al., 2000;Vandermosten et al., 2020). In line with this speculation, a meta-analysis found a greater reduction of brain activation in the left IPL in children with RD than in adults with RD in alphabetic languages (Richlan et al., 2011). Alternatively, the greater difference in children than adults with RD at the left IFG might be language-universal, since one study in English has found a positive age correlation in the left IFG in individuals with RD but not in typical readers (Shaywitz, et al., 2007), suggesting a delayed development in this region in RD readers. Moreover, activation in the left dorsal IFG has also been found to be related to phonological decoding in alphabetic languages (Cao et al., 2006;Klaus & Hartwigsen, 2019). Future research needs to examine whether it is the left IFG or the left temporoparietal area that shows greater differences in children than adults with RD in alphabetic languages.

In addition to the left IFG, we also found greater differences in children than adults with RD in the default mode network. We found a reduction of deactivation in the default mode network in children with RD but not in adults with RD during the auditory rhyming task, including the left medial frontal gyrus, left precuneus, right precentral gyrus, and right supramarginal gyrus. This finding suggests a deficiency in deactivating the default mode network during active tasks in children with RD, which eventually disappears in adulthood. Several developmental disorders have been found to be associated with abnormalities in the default mode network (Buchweitz et al., 2019;Cao et al., 2017;Finn et al., 2014), and our study further suggests that these abnormalities in the default mode network tend to be more evident in children than adults with RD.

### 4.3 Accumulative effects of RD

In the current study, we found greater abnormalities in adults than in children with RD for both the sentence reading fluency test and the function of the left ITG (VOI). Adults with RD showed a greater reduction of brain activation in the left ITG across all tasks than children with RD, suggesting an accumulative effect of RD, which means that the symptoms become more severe with time. This finding is consistent with a previous meta-analysis which found greater brain activation differences in the left fusiform gyrus in adults with RD than in children with RD (Richlan et al., 2011). Our brain-behavioral correlation analysis further showed a positive correlation between activation in the left ITG and sentence reading fluency scores in adults with RD, providing further evidence for the importance of the left ITG in fluent reading. Other studies have shown that the left ITG/fusiform gyrus is involved in rapid orthographic recognition, which is necessary in mature fluent reading (Dehaene et al., 2001), predicts future reading development (Centanni et al., 2019), and that activation in this region increases as reading fluency develops (Ozernov-Palchik et al., 2023), and as reading fluency demands increase (Benjamin & Gaab, 2012). This region has also been found to play a role in integrating orthography, phonology, and semantics (Purcell et al., 2014) and in making predictions during reading (Dambacher et al., 2009), which are essential for fluent reading. Our VOI analysis also suggested that the left ITG is more involved in orthographic processing than phonological processing. RD readers appear to never reach fluent orthographic reading by involving the left ITG to an appropriate degree.

The reason that adults with RD had greater abnormalities than children with RD in the left ITG is because typical readers had a greater developmental increase than RD readers in this region. Therefore, the discrepancy between RD readers and typical readers is enlarged with development, suggesting an accumulative effect of RD. Typical readers showed a developmental increase in the left ITG while RD readers showed a developmental increase in the left IFG as suggested in the VOI by age by group interaction. This is consistent with the reading development model which argues that there is a shift from phonological reading to orthographic reading in typical readers. However, RD readers did not follow the same route. It seems that their phonological reading develops in the left IFG, even though it takes much longer than typical readers; however, they never reach orthographic reading in the left ITG. This suggests that the obstacle in reading development in individuals with RD is to shift from phonological reading to orthographic reading.

This finding is also consistent with previous studies.Shaywitz et al. (2007)showed exactly the same finding in English readers with RD (7–18 years), which is age-related increase in the left ITG in typical readers and age-related increase in the left IFG in RD readers. Another study also showed that the left IFG represents a skill effect while the left ITG represents a deviant in RD (Cao et al., 2020). Consistent finding from a recent study on children (8–11 years) also showed that connectivity from the IPL to ITG was an RD effect while the connectivity from the ITG to IFG was a reading level effect (Di Pietro, Willinger, et al., 2023). However, it does not mean that the left OT’s function cannot be improved in RD readers. As demonstrated in previous intervention studies, the left OT area showed significant increases in activation after phonological intervention (Heim et al., 2015), morphological intervention (Richards et al., 2006), as well as executive function and attention intervention (Horowitz-Kraus et al., 2019). Activation increases in the left OT was also positively correlated with behavioral improvement (Nugiel et al., 2019;Romeo et al., 2018). Participants in the current study were those who never received intensive interventions, and that may be why there is an enlarged difference in the left OT with age. It implicates the importance of providing reading intervention for RD readers. Taken together, the major finding of the current study is that children with RD showed greater abnormalities in the dorsal phonological reading pathway in the left IFG, and adults with RD showed greater abnormalities in the ventral orthographic reading pathway in the left ITG.

In addition, in the whole-brain interaction effects of group by age, we found greater abnormalities in adults than in children with RD in several regions, including the bilateral STG in the auditory rhyming task. This is because in the bilateral STG during the auditory rhyming task, typical readers showed developmental increases, while RD readers showed no change or even developmental decreases. This is consistent with a recent study that found a lack of developmental change in the posterior STG in poor readers (Di Pietro, Karipidis, et al., 2023). The bilateral STG is essential for performing phonological calculations in the auditory rhyming task; therefore, typical adults showed greater activation than typical children, suggesting a greater task specialization in adults than in children (Booth et al., 2001). However, RD readers showed no developmental increase in task specialization in this region, suggesting that learning and development in RD readers may not follow the interactive specialization hypothesis (Johnson & Munakata, 2005), in which it is proposed that skill acquisition is associated with increased specialization of key brain regions and increased interactivities between regions in the relevant network.

Taken together, this is the first study that examined age-related changes in the brain functional differences in individuals with RD in Chinese. We identified the left IPL as the neural signature of RD, the left IFG as a difference in children with RD, and the left ITG as a difference in adults with RD. Future research with a longitudinal design would be desired to examine whether the same pattern holds. Research in other languages is also needed to examine whether these findings are universal across languages.

## 5 Limitation

One limitation of the current study is that the sample of RD children for the VR task was different from the sample for the AR and the VS task. This may have constrained our ability to identify common RD effects across different tasks, even though we have identified the left IFG and the left ITG as task-general RD effects in children and adults, respectively.

## 6 Conclusion

From a developmental perspective, we highlight the signature role of persistent phonological deficits associated with the left IPL in Chinese readers with RD, which may be related with causes of RD. Poor phonological decoding and reduced activation in the left IFG are more evident in children than adults with RD, while poor reading fluency and reduced activation in the left ITG tend to be accumulative effects of RD, which is more evident in adults than children with RD. Our findings provide important insights into understanding the etiology and prognosis of RD, and it implicates that reading treatment may consider a focus on boosting phonological decoding in children and reading fluency in adults with RD.

## Supplementary Material

Supplementary Material

## Data Availability

Data and scripts used in this study are available athttps://github.com/yanfeifei2008/Developmental_features_in_RD.
