# Supplementary material for: Age-related changes in individuals with and without reading disability: Behavioral and fMRI evidence
Source: Imaging Neurosci (Camb). 2024 Jul 15;2:imag-2-00232. doi: 10.1162/imag_a_00232 (PMC12272204; doi:10.1162/imag_a_00232)
Supplement: Supplementary Material [file imag_a_00232-supp.pdf]

Supplementary materials

Methods

fMRI tasks

In each task, there were 96 lexical trials, 24 perceptual trials and 48 null trials. For the lexical trials, there were four different conditions with 24 trials per condition. There was an O+P+ condition, in which the second characters of the prime and the target word had similar orthography by sharing a radical and rhymed (for example, ‘弥补/bu3/’, ‘纯朴/pu3/’); an O+P- condition, in which the second characters of the prime and the target word had similar orthography by sharing a radical but did not rhyme (for example, ‘资料/liao4/’, ‘学科/ke1/’); an O-P+ condition, in which the second characters of the prime and the target word rhymed but had different orthography (for example, ‘环保/bao3/’, ‘大炮/pao4/’); and an O-P- condition, in which the second characters of the prime and the target word had different orthography and did not rhyme (for example, ‘压缩/suo1/’, ‘傍晚/wan3/’). For the rhyming judgment, participants were asked to ignore the tone of the character, and half of the rhyming trials had the same tone and half had different tones. All of the four lexical conditions were included in the data analysis. Each task was divided into two runs with 6 minutes and 44 seconds per run.

Results

Performance on the tasks in the scanner

In order to take the trade-off between accuracy and reaction time into account, we calculated corrected reaction time by dividing the original reaction time by accuracy for each participant (Figure S1). We conducted a group × age × task

ANOVA separately for accuracy and reaction time (corrected). For accuracy, we found a significant interaction between group and task ($F(2, 301)=4.31$, $p=0.014$, $\eta^2=0.013$), which was driven by lower accuracy in RD than ACs for the auditory rhyming task ($F(1, 309)=58.63$, $p<0.001$, $\eta^2=0.163$) and the visual rhyming task ($F(1, 309)=17.94$, $p<0.001$, $\eta^2=0.050$), but not for the visual spelling task ($F(1, 309)=0.24$, $p=0.627$, $\eta^2=0.001$). We found a significant interaction between age and task ($F(2, 301)=8.89$, $p<0.001$, $\eta^2=0.028$), which was driven by greater developmental increase in accuracy for the auditory rhyming task ($F(1, 309)=44.17$, $p<0.001$, $\eta^2=0.130$) than the visual rhyming task ($F(1, 309)=5.15$, $p=0.001$, $\eta^2=0.050$), and the visual spelling task ($F(1, 309)= 5.16$, $p=0.024$, $\eta^2=0.001$). We found a significant interaction between age and group ($F(1, 301)=6.42$, $p=0.012$, $\eta^2=0.010$), which was driven by greater reduction of accuracy in children with RD compared to ACs ($F(1, 310)=50.19$, $p<0.001$, $\eta^2=0.147$) than in adults with RD compared to ACs ($F(1, 310)=8.20$, $p=0.004$, $\eta^2=0.196$). In addition, we also found a significant main effect of group ($F(1, 301)=85.76$, $p<0.001$, $\eta^2=0.133$) with RD readers having lower accuracy than ACs, a significant main effect of age ($F(1, 301)=63.05$, $p<0.001$, $\eta^2=0.098$) with children having lower accuracy than adults, as well as a significant main effect of task ($F(2, 301)=78.99$, $p<0.001$, $\eta^2=0.246$) with higher accuracy on the visual spelling task than the visual rhyming and auditory rhyming tasks, and higher accuracy on the visual rhyming task than the auditory rhyming task.

For reaction time, we found a significant interaction of group by age by task ($F(1, 301)=3.90$, $p=0.021$, $\eta^2=0.014$). Simple effect analysis showed that ACs were faster than RD readers in the auditory rhyming task only for adults ($F(1, 306)=14.61$, $p<0.001$, $\eta^2=0.043$), but not for children ($F(1, 306)=0.93$, $p=0.335$, $\eta^2=0.002$); ACs were faster than RD readers in the visual spelling task only for children ($F(1, 306)=10.79$, $p=0.001$, $\eta^2=0.032$ for adults), but not for adults ($F(1, 306)=1.20$, $p=0.274$, $\eta^2=0.004$). There were no difference between ACs and RD readers in the visual rhyming task ($F(1, 306)=0.01$, $p=0.951$, $\eta^2=0.001$ for children; $F(1, 306)=0.59$, $p=0.444$, $\eta^2=0.002$ for adults). In addition, We also found significant main effect of

group ($F(1, 301)=10.25, p=0.002, \eta^2=0.018$) with RD readers slower than ACs, a significant main effect of age ($F(1, 301)=82.44, p<0.001, \eta^2=0.147$) with children slower than adults, as well as a main effect of task ($F(2, 301)=74.98, p<0.001, \eta^2=0.268$) with shorter RT on the visual spelling task than the visual rhyming and auditory rhyming tasks, and shorter RT on the visual rhyming task than the auditory rhyming task.

Conjunction of age effects in typical and RD readers

For the auditory rhyming task, conjunction analysis showed common developmental increases in typical and RD readers in the left IFG, left STG, left ITG, right medial frontal gyrus, right precentral gyrus, right precentral gyrus, right STG, and right STG; common developmental decreases were found in the left inferior occipital gyrus, right medial frontal gyrus, right middle frontal gyrus, and right lingual gyrus (Table 2, Figure 3). For the visual rhyming task, conjunction analysis across typical and RD readers showed common developmental increases in the left IFG, left IFG, left ITG, left inferior occipital gyrus, and right inferior occipital gyrus (Table 2, Figure 3). For the visual spelling task, conjunction analysis across typical and RD readers showed common developmental increases in the left IFG, left superior parietal lobule, left fusiform gyrus, left middle occipital gyrus, right lingual gyrus, and right middle occipital gyrus. A common developmental decrease was found in the right middle frontal gyrus for the visual spelling task (Table 2, Figure 3).

VOI results

We also found a significant main effect of group ($F(1, 300) = 36.15, p <.001, \eta^2 =.057$) with RD readers lower than ACs, a significant main effect of age ($F(1, 300) = 50.01, p <.001, \eta^2 =.079$) with children lower than adults, as well as a significant main effect of task ($F(2, 300) = 14.34, p <.001, \eta^2 =.046$) with lower activation in the auditory rhyming task than in the visual rhyming task and the visual spelling task.

Verification analyses - Psychophysiological interaction (PPI) results

To understand why different abnormal patterns occurred in children and adults with RD, we performed psychophysiological interaction (PPI) analyses with the left IFG and left ITG as seed regions (Figure S7). Notably, we report only significant interactions between group and age here from the ANCOVA of group (AC, RD) by age (children, adults) with accuracy on the in-scanner task as the covariate.

For the auditory rhyming task, analysis with the seed in the left IFG revealed a significant group by age interaction in the right cingulate gyrus (peak: 4, -34, 32, cluster: 565 voxels), right IFG (peak: 56, 22, 18, cluster: 340 voxels), right middle frontal gyrus (peak: 38, 10, 40, cluster: 189 voxels), and right inferior parietal lobule (peak: 50, -46, 56, cluster: 436 voxels). Simple effect analysis showed that the interactions in the right cingulate gyrus and the right middle frontal gyrus were driven by reduced connections in children with RD but not adults with RD. The interaction in the right inferior parietal lobule was driven by reduced connections in children with RD and increased connections in adults with RD. The interaction in the right IFG was driven by increased connections in adults with RD but no difference in children with RD. No interactions were found when the left ITG was the seed region.

For the visual spelling task, analysis with the seed in the left IFG revealed a significant group by age interaction in the left middle frontal gyrus (peak: -22, 18, 14, cluster: 116 voxels), which was driven by reduced connections in children with RD but not in adults with RD. For the seed in the left ITG, we found a significant interaction for the right cingulate gyrus (peak: 20, -32, 44, cluster: 184 voxels), which was driven by increased connection in children with RD and reduced connection in adults with RD.

We did not find significant interactions for the visual rhyming task.

To summarize, PPI results showed that children with RD had reduced connections with the left IFG but adults with RD had normal or increased connections in the left IFG, and the opposite pattern for the left ITG.

Table S1. Demographic information for participants in the fMRI experiments.

Tasks	N				Age			
	CAC	CRD	AAC	ARD	CAC	CRD	AAC	ARD
Auditory rhyming	17	40	23	35	11.33±0.47	11.15±0.42	20.78±2.37	19.91±1.22
Visual rhyming	19	18	19	29	11.46±0.44	11.33±0.49	20.63±1.98	19.72±1.16
Visual spelling	18	38	23	34	11.35±0.46	11.16±0.41	20.78±2.37	19.88±1.23

CAC, age control children; CRD, children with RD; AAC, age control adults; ARD, adults with RD.

Table S2. The main effect of group and age separately for each task.

Brain regions	H	BA	Voxels	MNI Coordinate			Z
**The main effect of group**							
**Auditory rhyming**							
Inferior temporal gyrus	L	20/37	733	-48	-58	-16	5.32
Inferior frontal gyrus	L	6/8/9	640	-40	8	24	5.12
Inferior frontal gyrus	L	46	632	-46	30	10	4.80
Precentral gyrus	R	4/6	334	36	-18	54	4.75
Inferior parietal lobule	L	40	149	-48	-42	34	4.02
Superior parietal lobule	L	7	168	-28	-64	58	3.57
**Visual rhyming**							
Inferior temporal gyrus	L	20/37	202	-48	-54	-12	4.22
**Visual spelling**							
Insula	L	13	103	-34	-20	14	4.96
Precuneus	R		108	18	-48	10	4.29
Lingual gyrus	R	18	162	14	-90	-8	3.97
Middle temporal gyrus	L	19/37	112	-46	-62	-2	3.45
**The main effect of age**							
**Auditory rhyming**							
Precentral gyrus	R	3/4/6/13/22	5185	32	-24	54	7.63
Inferior frontal gyrus	L	4/6/8/9/46	1974	-44	26	18	6.23
Inferior temporal gyrus	L	20/37	396	-50	-50	-14	6.21
Inferior parietal lobule	L	40	408	-52	-48	38	5.30
Middle frontal gyrus	R	6/8/9	1328	38	32	38	5.28
Superior temporal gyrus	L	6/21/22/41/42	1179	-60	-18	6	5.22
Supramarginal gyrus	R	2/13/40	1066	58	-38	40	5.03
Middle frontal gyrus	L	8/9	593	-38	34	44	5.02
Fusiform Gyrus	L	18/19/37	1024	-36	-58	-16	4.95
Inferior frontal gyrus	R	44	93	52	12	4	4.51

Anterior cingulate gyrus	L	535	535	-10	36	26	4.35
Lingual gyrus	R	18/19	684	26	-84	-10	4.29
Inferior occipital gyrus	R	37	131	42	-64	-14	3.98
Inferior frontal gyrus	R	46	107	50	32	18	3.97
Superior parietal lobule	L	7	242	-26	-58	42	3.94
Superior temporal gyrus	L	38	91	-54	14	-14	3.89
Angular gyrus	L	39/40	203	-44	-60	48	3.88
Putamen	R		85	32	-2	-2	3.84
Angular gyrus	R	39	105	36	-68	48	3.48
Superior frontal gyrus	R	10	178	20	58	20	3.46
**Visual rhyming**							
Inferior frontal gyrus	L	6/8/9/46	1575	-44	28	18	6.42
Inferior temporal gyrus	L	18/19/37	1537	-52	-52	-12	6.24
Inferior parietal lobule	L	7/40	975	-24	-64	44	6.14
Inferior occipital gyrus	R	18/19	321	38	-86	-10	5.67
Middle frontal gyrus	R		298	54	36	20	5.34
Middle temporal gyrus	R	2/3/4/13/22/40	1421	48	-46	18	5.24
Superior occipital gyrus	R	17/18	322	22	-92	6	4.87
Inferior frontal gyrus	R	22/44	165	50	10	4	4.72
Middle temporal gyrus	L	22/39/40	329	-56	-62	22	4.61
Middle frontal gyrus	R	8/9	128	32	36	38	3.56
**Visual spelling**							
Lingual gyrus	R	7/18/19/39	2647	16	-84	-6	6.64
Precentral gyrus	R	3/4/6	852	34	-22	52	6.45
Inferior temporal gyrus	L	7/17/18/19/31/39	4530	-50	-52	-14	5.92
Supramarginal gyrus	L	40	302	-62	-44	34	5.63
Middle frontal gyrus	R	9	324	38	22	40	5.33
Supramarginal gyrus	R	40	552	54	-40	40	5.11
Middle frontal gyrus	R	6	144	44	10	50	4.94

Inferior frontal gyrus	L	6/9/46	818	-42	26	18	4.54
Insula	R	13	185	32	32	6	4.50
Cingulate gyrus	R	6/24	153	6	-10	50	3.97
Superior frontal gyrus	R	8	95	28	28	54	3.95
Middle frontal gyrus	L	9	158	-32	34	34	3.83
Inferior frontal gyrus	R	46	86	56	36	12	3.79

Table S3. Differences between typical readers and RD readers in adults and children separately for each task.

Brain regions	H	BA	Voxels	MNI Coordinate			Z
			**Adults AC>RD**				
**Auditory rhyming**							
Supramarginal gyrus	L	40	172	-62	-34	30	4.80
Superior temporal gyrus	L	41	395	-42	-38	28	4.74
Inferior temporal gyrus	L	37	743	-50	-58	-16	4.68
Hippocampus	L		162	-24	-6	-18	4.17
**Visual rhyming**							
Inferior temporal gyrus	L	37	245	-50	-54	-12	4.14
**Visual spelling**							
Inferior temporal gyrus	L	37	242	-48	-60	-6	4.03
			**Adults RD>AC**				
-	-	-	-	-	-	-	-
			**Children AC>RD**				
**Auditory rhyming**							
Cerebellum	L		2936	-10	-30	-20	5.91
Inferior frontal gyrus	L	6/46	2973	-42	38	14	5.23
Supplementary motor area	L	32	571	-2	14	46	5.08
Insula	R	13	292	34	30	0	4.60
Calcarine	R	18	164	24	-70	16	4.41
Caudate	R		248	22	8	20	3.92
Inferior temporal gyrus	L	37	146	-48	-60	-18	3.59
Superior parietal lobule	L	7	177	-18	-64	44	3.51
**Visual rhyming**							
Inferior frontal gyrus	L	44	261	-54	10	20	4.49
Lingual gyrus	L	17/18	265	-4	-70	8	4.25
Thalamus	L		262	-8	-18	-4	4.11
Putamen	L		172	-14	10	2	4.03

Lingual gyrus	L	19	144	-14	-52	-10	3.74
**Visual spelling**							
Lingual gyrus	L	18	263	-22	-76	-10	5.46
Insula	L	13	134	-32	-22	16	5.05
Caudate	L		202	-2	4	4	4.74
Thalamus	L		134	-10	-6	6	4.10
Fusiform	R	19	198	26	-74	-8	3.98
Inferior frontal gyrus	L	9	166	-30	4	30	3.95
Precentral gyrus	L	4	141	-44	-18	44	3.93
Calcarine	R	30	157	20	-48	12	3.82
		**Children RD>AC**					
**Auditory rhyming**							
Precentral gyrus	R	3/4	929	36	-20	56	6.98
Cingulate gyrus	L	7/31	2212	-10	-48	28	4.74
Putamen	R		208	30	-2	-4	3.78
Supramarginal gyrus	R	40	348	54	-44	30	3.69
**Visual rhyming**							
Medial frontal gyrus	L	10/32	267	-14	48	4	4.23
**Visual spelling**							
Precentral gyrus	R	3/4	499	34	-22	54	6.07
Cerebellum	L		156	-14	-54	-20	5.50

Table S4. Differences between adults and children in typical readers and RD readers in each task.

Brain regions	H	BA	Voxels	MNI Coordinate			Z
**AC Adults>Children**							
**Auditory rhyming**							
Precentral gyrus	R	3/4/6	1623	38	-22	62	inf
Inferior temporal gyrus	L	37	261	-50	-50	-14	5.41
Superior temporal gyrus	R	6/13/21/22	3159	60	-12	2	5.21
Supplementary motor area	R	6	410	4	-6	50	5.02
Superior temporal gyrus	L	22/41	1270	-58	-6	-2	4.66
Hippocampus	L		271	-26	-4	-18	4.49
Precuneus	L	31	325	-12	-54	26	4.37
Superior temporal gyrus	L	38	205	-50	8	-22	3.94
Inferior frontal gyrus	L	46	132	-40	28	18	3.90
**Visual rhyming**							
Inferior temporal gyrus	L	18/19/37	1127	-52	-52	-12	5.74
Inferior occipital gyrus	R	19	250	38	-86	-10	4.81
Inferior frontal gyrus	R	46	203	54	36	18	4.51
Inferior frontal gyrus	L	9	292	-52	14	32	4.23
Inferior frontal gyrus	L	46	384	-44	28	18	4.19
Inferior parietal lobule	L	40	147	-46	-42	42	4.11
**Visual spelling**							
Precentral gyrus	R	3/4	1003	34	-22	52	6.95
Inferior temporal gyrus	L	18/19/37	1356	-50	-52	-14	5.63
Cerebellum	L		126	-16	-50	-22	5.41
Lingual gyrus	R	17	256	16	-84	-4	5.32
Inferior temporal gyrus	R	18/19	948	50	-60	-12	4.85
Inferior temporal gyrus	R	20	117	54	-10	-28	4.46
Inferior frontal gyrus	L	9	156	-50	16	32	4.22

Superior frontal gyrus	L	9	140	-12	52	36	3.92
Insula	R	13	210	36	-16	18	3.92
Precuneus	L	31	373	-12	-46	44	3.91
Superior parietal lobule	L	7	188	-16	-72	54	3.75
Cingulate gyrus	R	6/24	133	4	-10	50	3.67
			**AC Children>Adults**				
**Auditory rhyming**							
Middle frontal gyrus	L		177	-32	32	24	4.27
Middle frontal gyrus	R	8/9	373	38	32	36	4.18
Anterior cingulate gyrus	R	32	135	10	30	24	4.11
Middle occipital gyrus	L	18/19	459	-38	-78	-2	4.08
Insula	R	47	141	32	20	-10	3.88
Inferior occipital gyrus	R		297	38	-76	-6	3.57
Middle frontal gyrus	L	10	137	-28	54	16	3.53
**Visual rhyming**							
Caudate	L		299	-10	8	8	4.55
**Visual spelling**							
Middle frontal gyrus	R	9	293	40	32	36	4.72
Inferior frontal gyrus	R	47	268	34	32	2	4.43
Putamen	L		470	-18	14	-8	4.07
			**RD Adults>Children**				
**Auditory rhyming**							
Inferior frontal gyrus	L	6/9/46	3377	-46	10	26	6.30
Supplementary motor area	L	6	708	-4	14	50	5.82
Putamen	R		449	-16	4	8	5.55
Inferior parietal lobule	L	7	632	-26	-58	42	4.94
Cerebellum	R		348	20	-64	-28	4.81
Superior temporal gyrus	R	22	456	60	6	-4	4.78
Caudate	R		361	24	30	18	4.64

Precentral gyrus	R	6/9	286	52	2	46	4.56
Middle frontal gyrus	R	6	130	24	-12	62	4.34
Middle temporal gyrus	L	22	186	-64	-18	0	4.26
Calcarine	L	31	244	-14	-74	22	4.21
Superior temporal gyrus	R		115	36	-48	2	4.00
Inferior temporal gyrus	L	37	172	-52	-60	-18	3.74
Inferior parietal lobule	L	40	163	-46	-40	46	3.68
**Visual rhyming**							
Inferior frontal gyrus	L	6/9/46	1546	-46	28	18	5.71
Middle occipital gyrus	L	7	833	-24	-60	34	5.48
Supplementary motor area	L	32	132	-4	16	48	4.91
Inferior temporal gyrus	L	37	296	-52	-50	-12	4.56
Inferior occipital gyrus	L	18	447	-18	-94	-6	4.34
Calcarine	R	17	269	14	-84	0	4.19
Inferior occipital gyrus	R	19	165	38	-84	-4	3.93
**Visual spelling**							
Precuneus	L	7/17/18/19	3455	-18	-64	52	5.40
Middle occipital gyrus	R	7/19	1611	34	-76	20	5.22
Lingual gyrus/Fusiform gyrus	R	17/18/19	654	18	-84	-10	5.18
Inferior frontal gyrus	L	6/9/46	1130	-48	26	20	4.37
			**RD Children>Adults**				
**Auditory rhyming**							
Supramarginal gyrus	R	40	2803	60	-34	42	5.86
Middle frontal gyrus	R	6/8/9	3900	44	12	52	5.66
Cingulate gyrus		31	1671	-8	-38	42	5.47
Supramarginal gyrus	L	39/40	1881	-56	-52	36	5.47
Inferior occipital gyrus	L	18/19	412	-46	-74	-10	4.61
Middle occipital gyrus	R	37	624	30	-82	4	4.20
Fusiform gyrus	L	37	129	-36	-48	-24	4.11

**Visual rhyming**							
Middle temporal gyrus	R	40	1756	50	-48	18	5.45
Superior temporal gyrus	L	22	140	-46	-2	-6	5.03
Middle temporal gyrus	L	22	273	-48	-58	14	4.94
Middle frontal gyrus	R	10	226	40	52	12	4.61
Cingulate gyrus	L	32	616	-6	44	6	4.45
Supramarginal gyrus	L		196	-56	-24	22	4.15
Superior frontal gyrus	R	10	210	16	54	18	3.92
Inferior frontal gyrus	R		338	50	10	0	3.92
Middle frontal gyrus	R	9	112	32	36	38	3.71
**Visual spelling**							
Supramarginal gyrus	L	40	286	-62	-44	34	5.42
Supramarginal gyrus	R	40	684	62	-42	22	4.60
Middle frontal gyrus	R	9	149	40	22	40	4.37
Cingulate gyrus	R	31	106	10	-28	42	4.10

Table S5. Brain activation in the conjunction analysis between comparisons with ACs and RCs in children and adults with RD for each task

Brain regions	H	BA	Voxels	MNI Coordinate		
**Adults AC>RD & RC>RD**						
**Auditory rhyming**						
Fusiform gyrus	L	37	49	-36	-46	-22
Inferior temporal gyrus	L	19	64	-50	-72	-10
Supramarginal gyrus	L		25	-48	-44	34
Supramarginal gyrus	L	40	80	-62	-34	30
**Visual rhyming**						
-	-	-	-	-	-	-
**Visual spelling**						
-	-	-	-	-	-	-
**Adults RD>AC & RD>RC**						
-	-	-	-	-	-	-
**Children AC>RD & RC>RD**						
**Auditory rhyming**						
Brainstem	L		52	-10	-28	-22
Cerebellum	R		93	8	-70	-26
Cerebellum	R		46	22	-70	-28
Cerebellum	R		21	32	-56	-29
Thalamus	L		254	-2	-20	-18
Inferior temporal gyrus	L	37	81	-50	-60	-18
Cerebellum	R		58	16	-52	-14
Lingual gyrus	R	18	28	5	-78	-7
Cerebellum	L		46	-8	-64	-8
Superior temporal gyrus	L	22	20	-48	8	-4
Inferior frontal gyrus	L	46	430	-32	22	4
Inferior frontal gyrus	R	13	152	34	30	2
Putamen	L		183	-12	14	2

Thalamus	L		65	-2	-12	10
Caudate	R		52	24	30	18
Middle frontal gyrus	L	10	114	-30	44	18
Superior occipital gyrus	R	18	40	22	-72	18
Inferior frontal gyrus	L	6/9	480	-42	8	26
Inferior frontal gyrus	L		22	-36	26	24
Supplementary motor area		6/32	408	10	22	46
Superior parietal lobule	L	7	39	-18	-64	46
Superior parietal lobule	L	7	20	-28	-62	54
**Visual rhyming**						
-	-	-	-	-	-	-
**Visual spelling**						
-	-	-	-	-	-	-
**Children RD>AC & RD>RC**						
**Auditory rhyming**						
-	-	-	-	-	-	-
**Visual rhyming**						
-	-	-	-	-	-	-
**Visual spelling**						
Precentral gyrus	R	4	364	38	-20	58

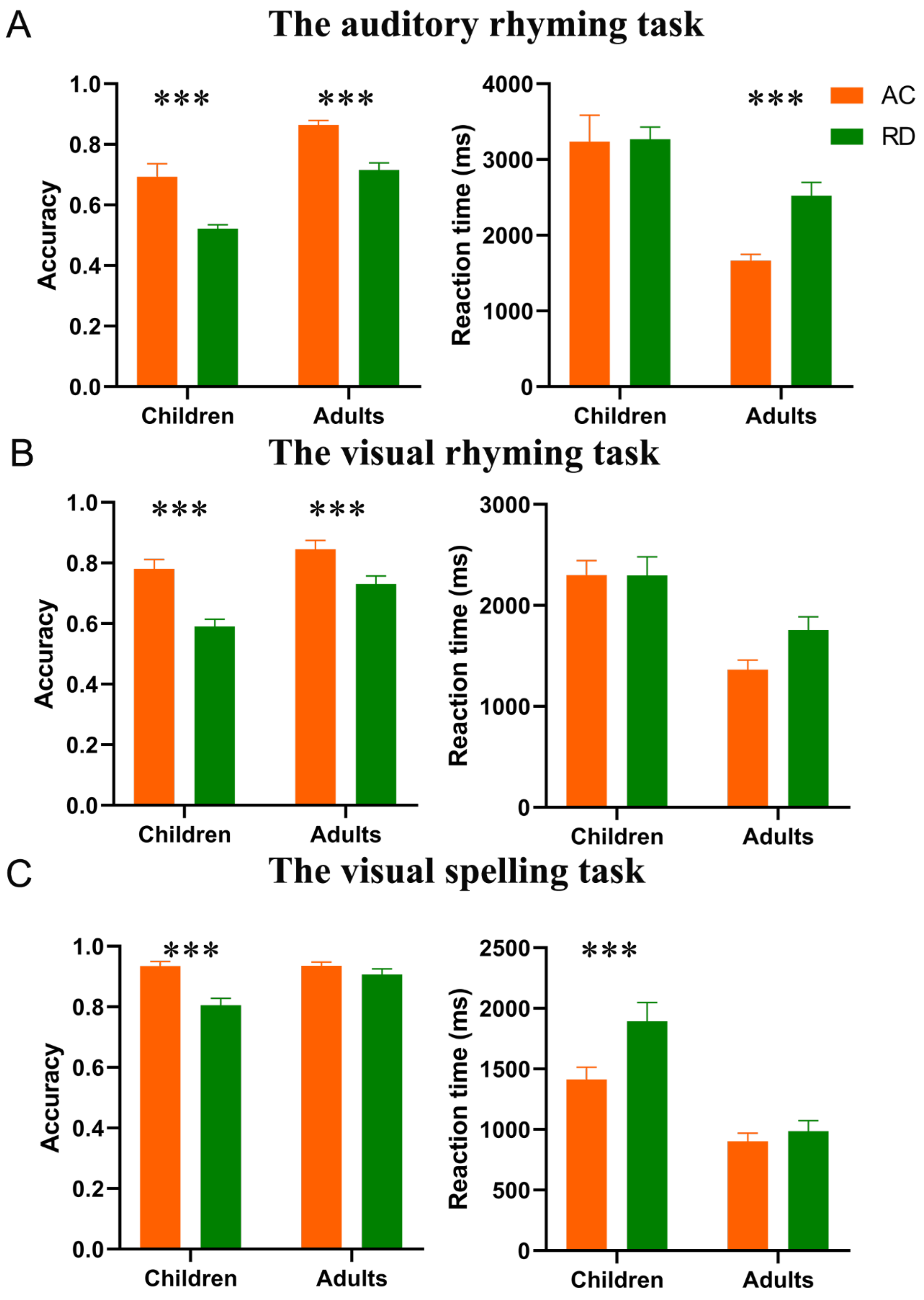

Figure S1. Accuracy and reaction time for the in-scanner tasks. *, $p<0.05$; **, $p<0.01$; *** $p<0.001$.

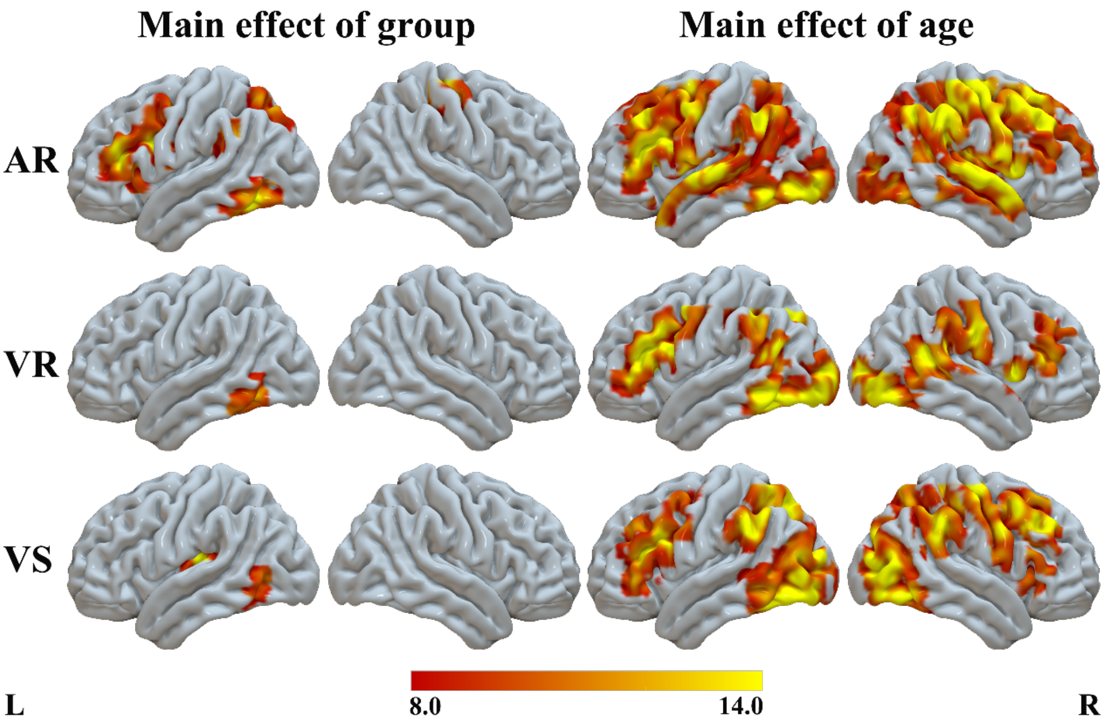

Figure S2. The main effects of group and age in the whole brain ANOVA for each task (*p*<0.005 uncorrected at the voxel level, FDR corrected *p*<0.05 at the cluster level).

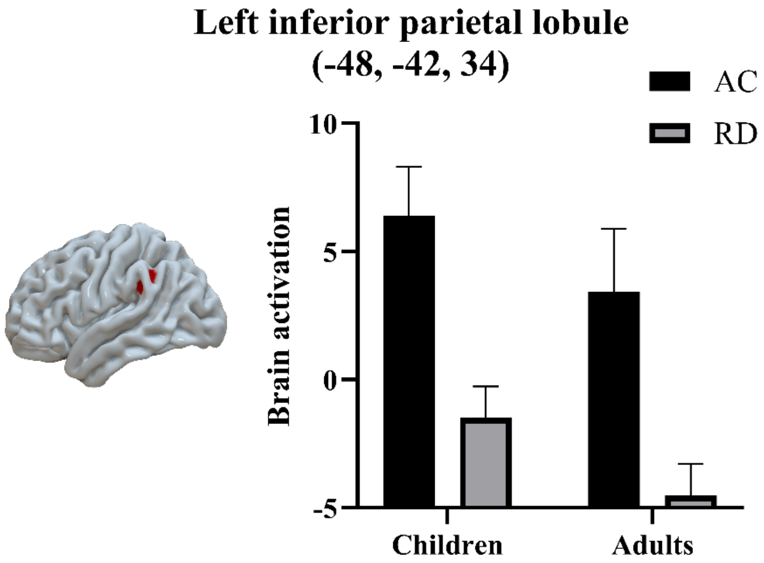

Figure S3. The overlap between children and adults in the main effect of group in the AR task when threshold was lowered. Both children with RD and adults with RD showed reduced activation in the left IPL than age controls.

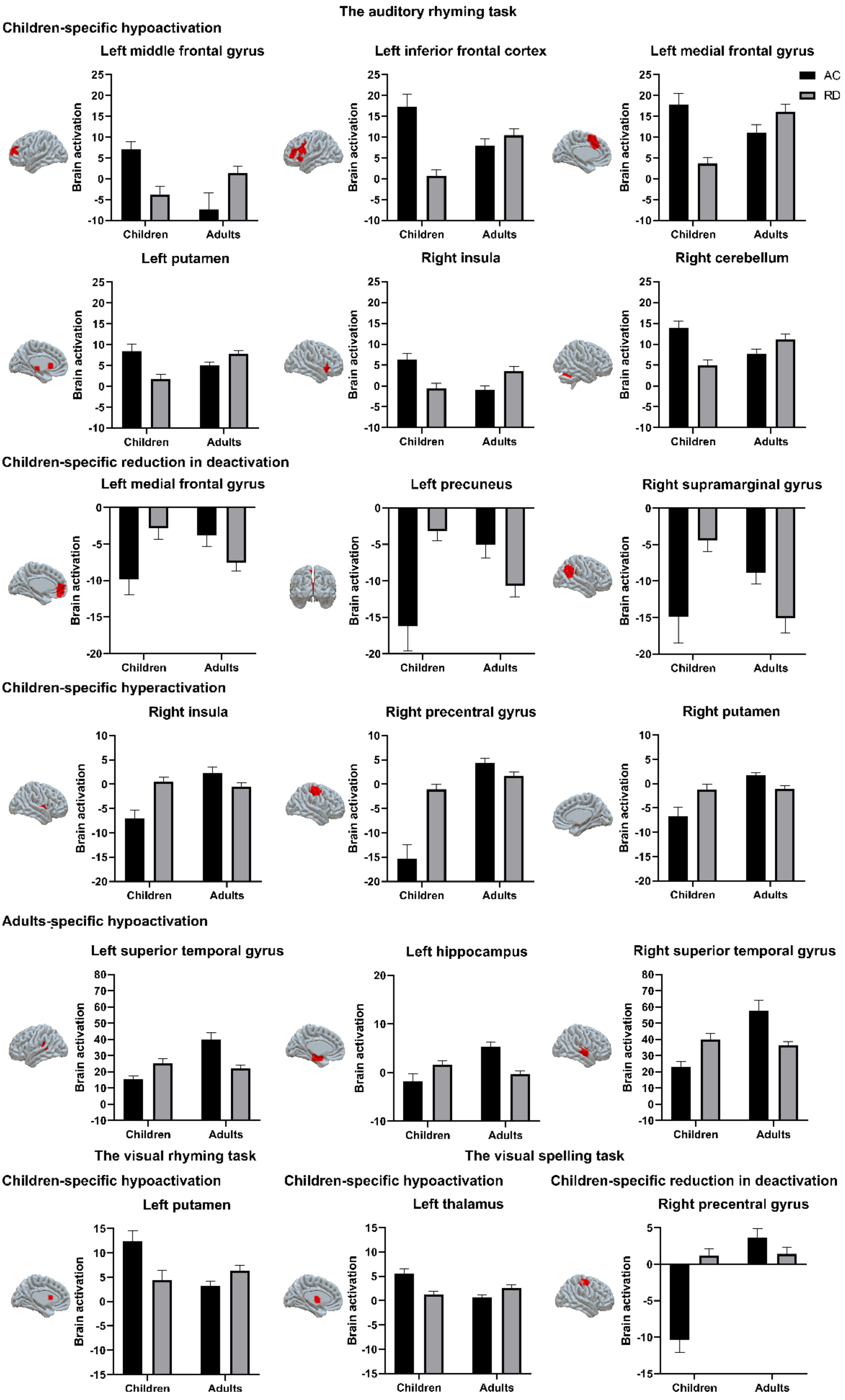

Figure S4. The significant interaction effects of group by age in the whole brain analysis for each task.

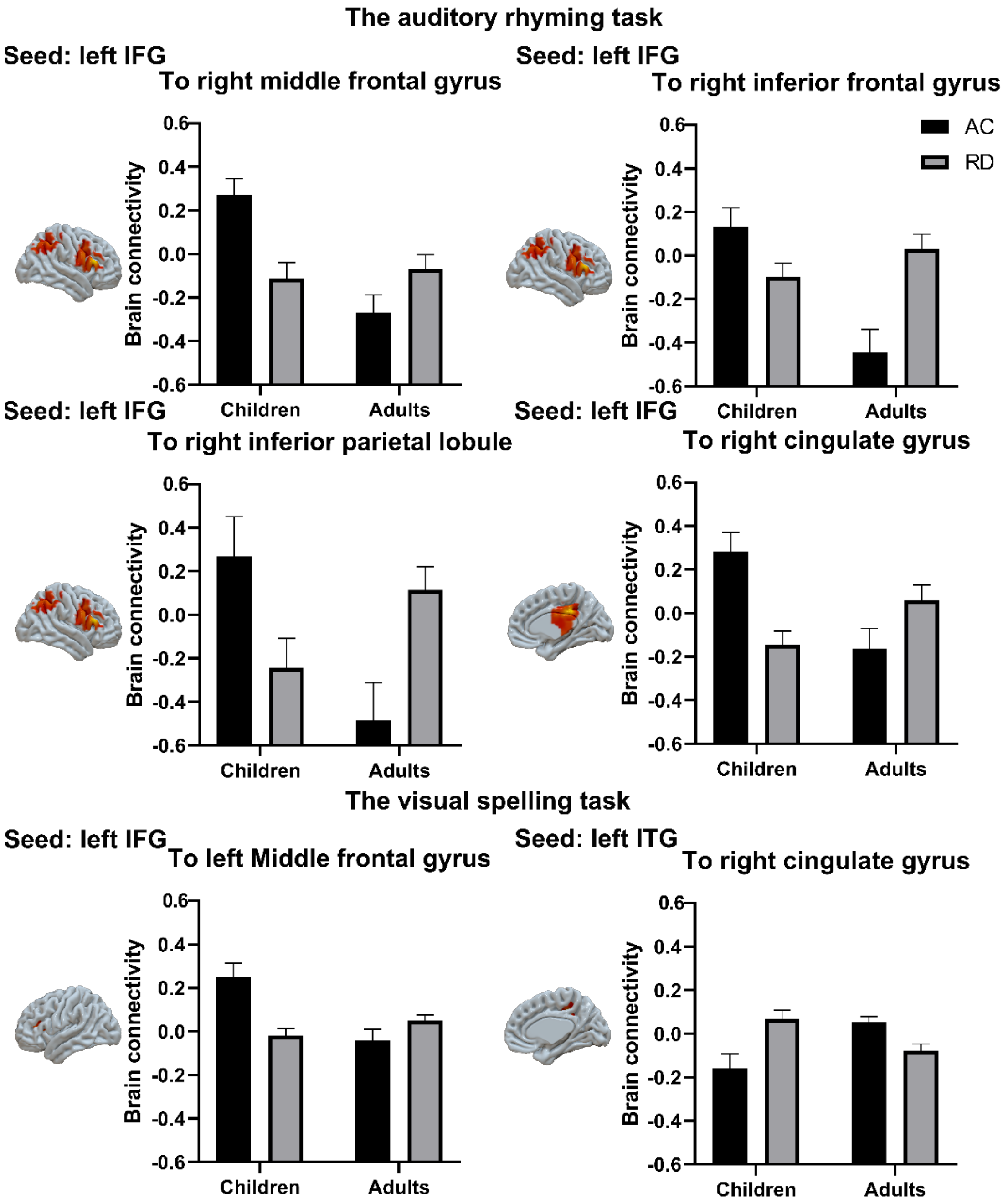

Figure S5. Significant interactions between group and age in the PPI analysis in each task for the seed regions of the left IFG and left ITG.
